# Transmission dynamics of ESBL/AmpC and carbapenemase-producing Enterobacterales between companion animals and humans

**DOI:** 10.3389/fmicb.2024.1432240

**Published:** 2024-09-03

**Authors:** Juliana Menezes, Siân-Marie Frosini, Scott Weese, Vincent Perreten, Stefan Schwarz, Andreia J. Amaral, Anette Loeffler, Constança Pomba

**Affiliations:** ^1^CIISA – Centre for Interdisciplinary Research in Animal Health, Faculty of Veterinary Medicine, University of Lisbon, Lisbon, Portugal; ^2^Associate Laboratory for Animal and Veterinary Sciences (AL4AnimalS), Lisbon, Portugal; ^3^Department of Clinical Science and Services, Royal Veterinary College, Hertfordshire, United Kingdom; ^4^Ontario Veterinary College, Guelph, ON, Canada; ^5^Division of Molecular Bacterial Epidemiology and Infectious Diseases, Institute of Veterinary Bacteriology, Vetsuisse Faculty, University of Bern, Bern, Switzerland; ^6^Institute of Microbiology and Epizootics, School of Veterinary Medicine, Freie Universität Berlin, Berlin, Germany; ^7^Veterinary Centre of Resistance Research, School of Veterinary Medicine, Freie Universität Berlin, Berlin, Germany; ^8^Science and Technology School, University of Évora, Évora, Portugal

**Keywords:** one health, ExPEC pathotypes, animal–human sharing, *Klebsiella pneumoniae*, *Enterobacter hormaechei* subsp. *hoffmannii*, CTX-M-15 ESBL, CTX-M-27, CMY-2

## Abstract

Antimicrobial resistance mediated by extended-spectrum beta-lactamase (ESBL)- and plasmid-mediated cephalosporinase (AmpC)-producing Enterobacterales, as well as carbapenemase-producing Enterobacterales have globally increased among companion animals, posing a potential health risk to humans in contact with them. This prospective longitudinal study investigates the transfer of ESBL/AmpC- and carbapenemase-producing Enterobacterales between companion animals and their cohabitant humans in Portugal (PT) and the United Kingdom (UK) during animal infection. Fecal samples and nasal swabs collected from dogs and cats with urinary tract infection (UTI) or skin and soft tissue infection (SSTI), and their cohabitant humans were screened for resistant strains. Relatedness between animal and human strains was established by whole-genome sequencing (WGS). ESBL/AmpC-producing Enterobacterales were detected in companion animals (PT = 55.8%; UK = 36.4%) and humans (PT = 35.9%; UK = 12.5%). Carbapenemase-producing Enterobacterales carriage was observed in one dog from Portugal (2.6%) and another dog from the UK (4.5%). Transmission of index clinical ESBL-producing *Escherichia coli* and *Klebsiella pneumoniae* strains to cohabitant humans was observed in three Portuguese households (6.9%, *n* = 43), with repeated isolation of the index strains on fecal samples from the animals and their cohabiting humans. In addition, longitudinal sharing of *E. coli* strains carried by companion animals and their owners was observed in other two Portuguese households and two households from the UK. Furthermore, a multidrug-resistant ACT-24-producing *Enterobacter hormaechei subsp. hoffmannii* strains were also shared within another Portuguese household. These results highlight the importance of the household as an epidemiological unit in the efforts to mitigate the spread of antimicrobial resistance, further emphasizing the need for antimicrobial surveillance in this context, capable of producing data that can inform and evaluate public health actions.

## 1 Introduction

The gastrointestinal tract is an important reservoir for antimicrobial-resistant Enterobacterales that may cause both intestinal and extraintestinal infections (Geurtsen et al., 2022). One critical mechanism driving antimicrobial resistance (AMR) in Enterobacterales involves the enzymatic inactivation of a wide range of beta-lactam antimicrobials, including third- and fourth-generation cephalosporins (3GCs and 4GCs) through the action of extended-spectrum β-lactamases (ESBLs) and plasmid-mediated cephalosporinases (AmpCs), and also carbapenem inactivation by carbapenemase-producing Enterobacterales (CPE) (Eiamphungporn et al., 2018). These medically important antimicrobials are critical in the management of serious infections caused by multidrug-resistant bacteria, and resistance to them poses a significant public health concern (World Health Organization, 2024).

Antimicrobial use can increase the burden of AMR, leading to long-term disturbance of commensal bacterial populations and prolonged carriage of antibiotic-resistant strains in the host environment even in the absence of selective pressure (Jernberg et al., 2010; Schmidt et al., 2018). In the endeavor to attenuate the emergence of resistance within the veterinary settings, the European Medicines Agency (EMA) has categorized 3GCs and 4CGs as ‘Restrict’ (Category B) in veterinary medicine and carbapenems as ‘Avoid’ (Category A) (EMA, 2019), the latter being recently classified as authorized only for use in humans by the World Health Organization (WHO), over concerns regarding the impact of the development of resistance in animals on humans (World Health Organization, 2024). However, the prevalence of ESBL- and AmpC-producing Enterobacterales (ESBL/AmpC-E) as well as CPE strains has been increasing worldwide in companion animals (Grönthal et al., 2018; Hong et al., 2020; Dazio et al., 2021).

ESBL/AmpC-E and CPE strains carried by companion animals are of particular One Health concern as they may act as a reservoir for self-infection and further transmit resistance genes and/or pathogens into other hosts including humans, other animals, and the environment (Damborg et al., 2016; Pomba et al., 2020; Donà et al., 2023). The close contact between humans and companion animals provides ample opportunities for the exchange of microbiota, potentially facilitating the spread of AMR (Pomba et al., 2020). Recent studies have shown that these bacteria can be transmitted between pets and their owners, indicating a bidirectional flow of resistant strains (Grönthal et al., 2018; Toombs-Ruane et al., 2020; Van Den Bunt et al., 2020). However, little is known about the dynamics of transmission in humans and companion animals belonging to the same household, particularly from sick companion animals to their healthy owners.

We hypothesize that companion animals under antibiotic treatment will share resistant bacteria with their cohabitant humans. As such, this study aims to explore the contribution of companion animals under antibiotic treatment in the AMR dissemination to the community setting. Hence, to assess the within-household transmission of resistant bacteria, a prospective and longitudinal study was conducted to describe the molecular epidemiology and evaluate the sharing of ESBL/AmpC-E and CPE strains between companion animals under antimicrobial treatment for skin and soft tissue infections (SSTI) or urinary tract infections (UTIs) and their cohabitant humans in the community. The sharing of ESBL/AmpC- and carbapenemase-producing strains was analyzed with two approaches, after the detection of their carriage among companion animals and their cohabitant humans: (i) the transmission of the animals’ clinical pathogenic strain to their cohabitant household humans and (ii) the transfer of resistant strains between infected companion animals under antibiotic therapy and their cohabitant humans, resulting in carriage and/or colonization. This was a 2-year, multicenter study conducted in Lisbon, Portugal, and the South-East United Kingdom (UK).

## 2 Materials and methods

### 2.1 Ethics approval

Ethical clearance for the collection of samples from humans and companion animals was obtained from the Faculty of Veterinary Medicine, University of Lisbon (FMV-ULisboa) Ethics Committee for Research and Education and the Royal Veterinary College (RVC) Ethics and Welfare Committee (ethics reference number: CEBEA 027/2018 and URN 2017 1750–3, respectively). Written consent for sample collection and the use of participants’ anonymized questionnaire-derived data was obtained from owners prior to enrollment in the study. Research was conducted in accordance with the Declaration of Helsinki and national and institutional standards.

### 2.2 Study design and setting

This study was part of an international prospective longitudinal observational study conducted at the Small Animal Veterinary Teaching Hospital of the Faculty of Veterinary Medicine, University of Lisbon, Portugal, and the RVC Small Animal Veterinary Referral Service, Royal Veterinary College, Hatfield, UK.

Between 2018 and 2021, dogs (*n* = 60) and cats (*n* = 5) that were presented to the veterinary hospitals for care for infection consultations were enrolled on a voluntary basis, together with their cohabitant humans (*n* = 102), if they fulfilled the criteria for diagnosis of the following infections: UTI according to the International Society of Companion Animal Infectious Diseases (ISCAID) guidelines (Weese et al., 2019), SSTI according to results of diagnostic tests (e.g., cytology and/or culture), and typical clinical signs of superficial pyoderma, deep pyoderma, and wound infections. Other inclusion criteria for enrollment of companion animals and humans were as follows: (i) no systemic antimicrobial therapy in the last 3 months; (ii) no topical antimicrobial therapy 2 days before sampling; and (iii) have lived together for at least 3 months (cohabitant). To ensure that participation was anonymous, households, humans, and animals were coded.

Epidemiological questionnaires assessing participants’ general health status, current or previous medical treatments, and exposure to healthcare facilities, were filled out by the human participants. In addition, owners were asked about their animal’s lifestyle, diet, contact with other animals, and closeness of the contact with their human counterparties, such as kissing/licking the owner’s face. For all variables on the questionnaire, the option ‘Prefer not to answer’ was available. The number of answers collected for any specific factor depended on whether the owner decided to disclose the information. Questionnaire variables and responses, by country, are listed in Table 1.

**Table 1:** Questionnaire responses on demographic, social, and clinical data of dogs (*n* = 60), cats (*n* = 5), and their cohabiting humans (*n* = 102) by country, 2018–2021.

Variables	Participants	Portugal		United Kingdom	
		Positive responses	Beta-lactam resistance carriage	Positives responses	Beta-lactam resistance carriage
**Demographic**					
Female	Humans	46 (*n* = 78)	15	6 (*n =* 11)	0
	Dogs	20 (*n* = 38)	14	3 (*n =* 7)	1
	Cats	2 (*n* = 5)	0	N/A	N/A
Male	Humans	32 (*n* = 78)	13	5 (*n =* 11)	2
	Dogs	18 (*n* = 38)	7	3 (*n =* 6)	2
	Cats	3 (*n* = 5)	3	N/A	N/A
Median age (years)	Humans	43.4 (*n* = 75)	N/A	47 (*n =* 13)	N/A
	Dogs	8.3 (*n =* 38)	N/A	6.5 (*n =* 7)	N/A
	Cats	8.8 (*n* = 5)	N/A	N/A	N/A
**Clinical**					
Antibiotic treatment 3 to 12 months before sampling	Humans	31 (*n* = 75)	15	4 (*n =* 13)	3
	Dogs	31 (*n* = 38)	17	14 (*n* = 22)	5
	Cats	4 (*n =* 5)	3	N/A	N/A
Antibiotic treatment 3 to 6 months before sampling	Humans	17 (*n =* 77)	9	3 (*n =* 12)	2
	Dogs	15 (*n =* 38)	8	2 (*n* = 18)	2
	Cats	0 (*n =* 5)	N/A	N/A	N/A
Hospitalization in the 12 months before sampling	Humans	6 (*n =* 77)	2	1 (*n =* 13)	0
	Dogs	14 (*n =* 38)	10	3 (*n* = 8)	2
	Cats	3 (*n =* 5)	2	N/A	N/A
The companion animal had probiotic treatment in the last year	Dogs	5 (*n =* 38)	4	2 (*n =* 8)	1
	Cats	1 (*n =* 5)	1	N/A	N/A
The companion animal had glucocorticoid treatment in the last year	Dogs	16 (*n =* 38)	6	N/A	N/A
	Cats	0 (*n =* 5)	N/A	N/A	N/A
The human has a chronic disease	Humans	29 (*n =* 74)	14	2 (*n* = 12)	0
**Social**					
The companion animal has an indoor lifestyle	Dogs	34 (*n =* 38)	20	11 (*n =* 13)	4
	Cats	5 (*n =* 5)	3	N/A	N/A
The companion animal socializes with other animals outside the household	Dogs	17 (*n =* 38)	8	7 (*n =* 13)	3
	Cats	0 (*n =* 5)	N/A	N/A	N/A
The companion animal participates in pet animal shows	Dogs	0 (*n =* 38)	N/A	0 (*n* = 13)	N/A
	Cats	0 (*n =* 5)	N/A	N/A	N/A
The companion animal is a working animal	Dogs	0 (*n =* 38)	N/A	0 (*n =* 13)	N/A
	Cats	0 (*n =* 5)	N/A	N/A	N/A
The companion animal stayed in a pet hotel in the last year	Dogs	6 (*n =* 38)	2	1 (*n =* 13)	0
	Cats	0 (*n =* 5)	N/A	N/A	N/A
The companion animal sleeps in the human bed	Dogs	14 (*n =* 38)	8	3 (*n =* 13)	0
	Cats	4 (*n =* 5)	3	N/A	N/A
The human cleans up companion animal waste	Humans	49 (*n =* 78)	27	13 (*n =* 13)	6
The human feeds companion animal	Humans	59 (*n =* 78)	35	13 (*n* = 13)	6
The human pets/cuddles with companion animals	Humans	71 (*n =* 78)	42	13 (*n* = 13)	6
The human allows kissing/ licking by companion animals	Humans	36 (*n =* 76)	20	13 (*n* = 13)	6
**Other**					
The companion animal eats raw meat/raw fish	Dogs	3 (*n =* 38)	2	2 (*n =* 13)	1
	Cats	0 (*n =* 5)	N/A	N/A	N/A
The human is a healthcare professional	Humans	8 (*n =* 78)	3	1 (*n =* 13)	0
The human traveled abroad in the past 12 months	Humans	35 (*n =* 76)	22	8 (*n* = 13)	2

Data are reported as the number of individuals; n indicates the total responses. For carriage results, data are reported as a number of positive participants who presented the characteristic. N/A, not applicable.

### 2.3 Sample collection

Samples were collected, immediately after enrollment (T0), 1 week after antimicrobial treatment started (T1), 1 month after antimicrobial treatment started (T2), and 2 months after antimicrobial treatment started (T3) (Table 2). At each timepoint, the inclusion criteria were reviewed, and participants were excluded if any change was reported. Epidemiological variables were also monitored to ensure that changes in these factors, such as household environment, that could be possible contamination sources, were accounted for throughout the study. Acquisition of follow-up samples depended on the owner’s willingness to continue to participate in the study with their respective companion animal. For these reasons, at T1, sample collection was performed only for 18 households in Portugal and only in one household in the UK, while for T2, 19 households from Portugal and 16 households from the UK were studied. Some participants did not deliver samples a week after the enrollment and returned to give samples a month later at T2 (Table 2).

**Table 2:** Data collection timepoints for the longitudinal study by country, 2018–2021.

Data collection timepoints	Portugal (*n* = 43 households)		United Kingdom (*n* = 22 households)	
	Animals (*n =* 43)	Humans (*n =* 78)	Animals (*n* = 22)	Humans (*n* = 24)
T0: recruitment	33	59	22	24
T1: antibiotic treatment^a,b^	18	41	1	1
T2: 1 month after T0^c^	19	37	16	17
T3: 2 months after T0	14	27	11	12

^aT1 was performed 1 week after antimicrobial treatment started.^

^b^For 10 households from Portugal (10 animals and 18 humans), recruitment started at this timepoint.

^c^For 10 households from Portugal (10 animals and 15 humans) and 15 households from the UK (15 animals and 17 humans), sample collection was not possible at T1 but only at T2.

At each time point, animals were evaluated by the attending veterinarian, and infection samples were collected and analyzed by the hospital’s Microbiology diagnostic laboratory according to standard procedures. In Portugal, the clinical strains were subsequently molecularly characterized for research purposes.

Instructions for at-home fecal sample collection were given to the owners. Human feces were to be collected directly into sterile plastic containers or by using ‘FeCol’ feces collection papers (Alpha Laboratories Ltd., United Kingdom) and then transferred into a sterile plastic container. Infant fecal samples were collected from diapers by their parents. Companion animal’s partial fecal samples (that did not touch the ground) were collected using sterile gloves and placed into a sterile container. Nasal swabs were collected by an attending veterinarian; in brief, a single sterile cotton swab was inserted approximately 1 cm into one nostril and then rotated along the mucous membrane for 5 s. The samples were stored for a maximum of 48 h at 4°C until processing.

### 2.4 Sample processing

One gram of homogenized fecal sample was added to 10 mL of sterile 0.85% NaCl (Merck, Germany) solution and mixed thoroughly by vortexing. Ten microliters of fecal suspension were plated onto MacConkey agar plates (Biokar Diagnostics, France) with and without 1.5 μg/mL of cefotaxime (Sigma-Aldrich, US) or 1 μg/mL meropenem (Sigma-Aldrich, US) supplementation; MacConkey agar plates containing temocillin 30μg disk (Mast Group, UK) were used as phenotypic indicator of OXA-48-like production (Da Silva et al., 2022). To improve the detection of low numbers of resistant bacteria, 1 g of feces was also diluted in 10 mL of sterile buffered peptone water (Biokar Diagnostics, France) and incubated at 36 ± 1°C for 24 h, followed by inoculation of 10 μL onto selective plates as described above. Nasal swabs were inoculated in 3 mL of NaCl 0.85% (Merck, Germany), and then 100 mL of this were streaked on selective media described above. After overnight incubation at 36 ± 1°C, up to five resistant suspected colonies of each different morphology were isolated and stored in 20% glycerol (Sigma-Aldrich, US) brain heart infusion broth (Biokar Diagnostics, France) at −20°C until processing.

### 2.5 Molecular characterization of isolates

Bacterial DNA was extracted by heat lysis and centrifugation for all obtained isolates (Féria et al., 2002) and species identification was performed by 16S rRNA gene sequencing, as described elsewhere (Salisbury et al., 1997). An extended PCR scheme described by Doumith et al. (2012) was used to assign the *Escherichia coli* isolates to one of the four major phylogroups (A, B1, B2, and D).

Genetic relationships between all *E. coli* isolates were initially determined by repetitive element sequence-based PCR (REP-PCR) typing (Versalovic et al., 1991). Persistence was defined as the isolation of strains with matching molecular profiles (REP-PCR and resistance determinants) from repeated samples of the same subject.

All isolates were tested for the presence of specific ESBLs (*bla*_CTX-M-type_, *bla*_TEM_, and *bla*_SHV_) (Eckert et al., 2006; Pomba et al., 2006), AmpC variants (*bla*_CIT-type_, *bla*_FOX-type_, *bla*_MOX-type_, *bla*_DHA-type_, *bla*_ACT-type_, and *bla*_MIR-type_) (Marques et al., 2019), and carbapenemases (*bla*_AIM_, *bla*_DIM_, *bla*_GIM_, *bla*_SIM_, *bla*_IMP_, *bla*_VIM_, *bla*_SPM_, *bla*_NDM_, *bla*_KPC_, *bla*_BIC_, and *bla*_OXA-48-like_) (Poirel et al., 2011) encoding genes by PCR and sequencing, due to their clinical relevance, prevalence in antimicrobial resistance, and major public health impact (Bevan et al., 2017; Salgado-Caxito et al., 2021; Da Silva et al., 2022; Zamudio et al., 2022; UK Health Security Agency, 2024).

### 2.6 Antimicrobial susceptibility testing

Minimum inhibitory concentrations (MICs) of a panel of antibiotics were determined by broth microdilution using the MicroScan® Neg MIC Panel Type 44 (Beckman Coulter, US) following guidelines by the European Committee on Antimicrobial Susceptibility Testing (EUCAST) clinical breakpoints 2024 (The European Committee on Antimicrobial Susceptibility Testing, 2024) and the Clinical and Laboratory Standards Institute (CLSI) (CLSI, 2024). Multidrug resistance was defined as non-susceptibility to at least one agent in three or more antimicrobial class categories (Sweeney et al., 2018). For *E. coli* strains, antimicrobial susceptibility testing was only performed for one representative isolate from each REP-PCR profile detected per participant per timepoint.

### 2.7 Whole-genome sequencing

ESBL/AmpC-producing Enterobacterales strains (*n* = 38) shared by companion animals and cohabitant humans based on REP-PCR profiling were further characterized by WGS. For this part of the study, genomic DNA was extracted using the NZY Tissue gDNA Isolation kit (NZYTech, Portugal). Library preparation was performed with the TruSeq DNA PCR-Free preparation kit and sequenced using the Illumina NovaSeq 6000 system with 2 × 150 bp paired-end reads (Illumina, San Diego, California, US) at a commercial company (Macrogen, Seoul, Republic of Korea).

The raw sequence reads were assessed for quality using FastQC v0.11.9 (Andrews, 2010) and filtered for low-quality reads using PRINSEQ v0.20.4 (Schmieder and Edwards, 2011) (mean base quality score of ≥20 and minimum read length of 90 nt), making an average of 9.1×106 high-quality reads per library. *De novo* assemblies were generated with SPAdes v3.14.1 (Bankevich et al., 2012) followed by two runs of polishing with Pilon v1.24 (Walker et al., 2014) and annotated using Prokka v1.14.6 (Seemann, 2014). Assemblies’ quality was assessed using QUAST v5.0.2 (Gurevich et al., 2013). Genome assemblies presented an average of L50 = 9.6 (range from 6 to 14) and N50 = 1.9×105 (range from 9.1×104 to 3.9×105) and an average depth of 258x; full WGS statistics are listed in Supplementary Table S1.

#### 2.7.1 Genome sequencing analysis

Antimicrobial resistance genes were assessed using AMRFinder (Feldgarden et al., 2021) and ResFinder (Bortolaia et al., 2020); plasmid replicons were obtained using PlasmidFinder (Carattoli et al., 2014), and sequence types were assigned using the MLST 2.0 (Larsen et al., 2012). *E. coli* virulence factors were obtained using VirulenceFinder 2.0 (Joensen et al., 2014), whereas *Klebsiella* spp. virulence factors were obtained using the Pathogenwatch web application (Centre for Genomic Pathogen Surveillance, 2018). *E. coli* strains were classified as extraintestinal pathogenic *E. coli* (ExPEC), uropathogenic *E. coli* (UPEC), or avian pathogenic *E. coli* (APEC) based on established molecular definitions (Menezes et al., 2023).

#### 2.7.2 Clonal relationships

Three datasets were defined for the possible shared 3GC-resistant strains, one including *E. coli* strains (*n* = 29), a second comprising *K. pneumoniae* strains (*n* = 7), and a final one with *Enterobacter hormaechei* strains (*n* = 2). Parsnp v1.2 (Treangen et al., 2014) was used to generate single nucleotide polymorphism (SNP) alignment. The complete genome sequence of *E. coli* K-12 MG1655 (GenBank accession: GCA_000005845.2) was used as the reference genome for the *E. coli* dataset, *K. pneumoniae* subsp. *pneumoniae* MGH 78578 (GenBank accession: GCA_000016305.1) as reference for *K. pneumoniae* dataset, and *E. hormaechei* NCTC 9394 (GenBank accession: GCA_000210775.1) as reference for *E. hormaechei* dataset. Gubbins (Croucher et al., 2015) and RaxML-NG (Kozlov et al., 2019) were used to provide a maximum likelihood tree with 100 bootstrap repeats based on the recombination core genome alignment. Snp-dists v.0.8.2 (Seemann et al., 2021) was used to extract the number of SNPs between strains. Microreact platform (Argimón et al., 2016) was used to visualize the phylogenetic tree linked to antimicrobial resistance data. Comparison of ORFs from companion animal’s-owner paired strains was performed by using the EasyFig and the BLASTn algorithm (Sullivan et al., 2011).

### 2.8 Statistical analysis

Statistical analysis was performed using the SAS statistical software package for Windows, version 9.3 (SAS Institute Inc., Cary, United States). For comparative analysis of baseline characteristics, Fisher’s exact test was used for categorical variables, and a *p*-value<0.05 was considered significant.

To identify risk factors for ESBL/AmpC-E carriage, contingency tables were generated using the collected demographic and clinical data to perform univariable logistic regression analysis. Since no variables with a *p*-value<0.1 were identified, it was not possible to build multivariable models.

## 3 Results

### 3.1 Study population

#### 3.1.1 Dogs and cats

A total of 43 households from Portugal and 22 from the UK were enrolled in this study (Table 2; Supplementary Figure S1), and each household had one companion animal.

In Portuguese households, 43 companion animals were included, consisting of 38 dogs and 5 cats. Among these animals, 21 had UTIs and 22 had SSTIs. In the UK households, 22 companion animals were included, all of which were dogs. Four of these dogs had UTIs, and 18 had SSTIs.

Table 1 presents detailed demographic, social, and clinical data for the study participants. The ages of the companion animals ranged from 1.8 to 15 years, with a median age of 8 years (*n* = 50). Most of the companion animals in both countries lived indoors. In Portugal, cats and dogs tend to sleep more often in their owners’ beds compared to dogs in the UK. Moreover, in Portugal, 81.6% (*n* = 31/38) of the dogs and 80% of the cats received antimicrobials within the 12 months before sampling, and for the UK dogs, this percentage was 63.6% (*n* = 14/22) (Table 1).

#### 3.1.2 Pet owners

In Portugal, a total of 78 humans cohabitating with companion animals with SSTI or UTI provided nasal and fecal samples. In the UK households, 24 humans were enrolled. The households had different compositions, with 1 to 5 humans per household. Human ages ranged from 3 months to 82 years, with a median age of 43.3 years (*n* = 88).

A high number of humans reported close contact behaviors with their companion animals, such as petting/cuddling and being kissed/licked by them. Regarding antibiotic treatments, among the humans who filled in the questionnaire, 41.3% (*n* = 31/75) from Portugal and 30.8% (*n* = 4/13) from the UK took antibiotics within the 12 months before sampling (Table 1).

### 3.2 Prevalence of ESBL/AmpC- and carbapenemase-producing Enterobacterales carriage

Considering all four sampling timepoints, CPE strains were recovered from only two dogs (3.3, 95%CI, 0–8, *n* = 2/60), specifically one from Portugal (2.6, 95%CI, 0–7.9, *n =* 1/38) and the other from the UK (4.6, 95%CI, 0–13.9, *n =* 1/22).

ESBL/AmpC-E strains were isolated from 30.4% (95%CI, 21.3–39.5, *n =* 31/102) humans and 49.2% (95%CI, 36.7–61.7, *n* = 32/65) companion animals (dogs and cats) in at least one timepoint sample. There was no significant difference between the prevalence of ESBL/AmpC-E carriage in humans and companion animals (*p* = 0.158). In addition, no significant difference was found between carriage in animals with SSTI or UTI (*p* = 0.060); hence, these infection groups were combined for further analysis.

#### 3.2.1 Comparative analysis by country

When comparing the two countries, no significant difference was found in the prevalence of ESBL/AmpC-E strains in companion animals from Portugal (55.8, 95%CI, 40.3–71.3, *n =* 24/43) and the United kingdom (36.4, 95%CI, 14.5–58.2, *n =* 8/22) (*p* = 0.138).

For humans, a significant difference was detected with a higher prevalence of ESBL/AmpC-E strains in Portugal (35.9, 95%CI, 25–46.8, *n =* 28/78) than in the United kingdom (12.5, 95%CI, 0–26.8, *n =* 3/24; *p* = 0.029). Due to this variation in results according to the country, the risk factor analysis for both companion animals and humans was carried out separately for each country.

Table 1 shows the complete set of variables considered in the risk factor analysis. None of them were significantly associated with ESBL/AmpC-E or CPE carriage in humans or companion animals (*p*-values>0.1).

#### 3.2.2 Isolate distribution

In total, 254 ESBL/AmpC-E and/or CPE carriage isolates were recovered from 63 participants (31 humans and 32 companion animals). These comprised two *Enterobacter hormaechei* subsp. *hoffmannii* isolates, 23 *K. pneumoniae,* and 229 *E. coli* isolates. REP-PCR profiling showed that only 115 *E. coli* strains were non-duplicate (Supplementary Figures S2-S4: dendrograms generated from REP-PCR fingerprinting of PT and UK strains). Among these non-duplicate *E. coli* isolates, phylogroup A and B1 were most frequently found in Portugal, followed by B2, while in the UK, phylogroup D was the most common (Supplementary Table S2).

#### 3.2.3 Antimicrobial susceptibility

Enterobacterales non-duplicate strains (*n* = 140) were further characterized through antimicrobial susceptibility testing, and MICs for each strain are displayed in Supplementary Table S2. All the strains were resistant to cefotaxime, a 3GC agent, presenting MIC values for this antibiotic ranging from 4 to >32 mg/L. Among these, two strains from a Portuguese dog were recovered from MacConkey agar plates supplemented with temocillin (30 μg) antibiotic disks and were susceptible to meropenem (MIC values ≤1 mg/L). Other four strains from a UK dog were recovered from meropenem-supplemented MacConkey agar plates, presenting a resistant profile to this antibiotic (MIC values >8 mg/L).

In Portugal, high resistance rates among companion animal strains were also observed for ampicillin (94.8%), the combination of ampicillin/sulbactam (84.5%), fourth-generation cephalosporin, cefepime (75.9%), and ciprofloxacin (63.8%). Human strains showed similar resistance patterns, with all of them being resistant to ampicillin and 88.7% to cefepime. Notably, a large percentage of carriage strains from companion animals were multidrug-resistant (96.2%), while 66% of the human strains presented this profile (Table 3).

**Table 3:** Antimicrobial resistance of ESBL/AmpC- and Carbapenemase-producing Enterobacterales strains isolated from feces and nasal swabs of companion animals and their cohabitant humans in Portugal and the United Kingdom.

Antimicrobial	Portugal		United Kingdom	
	Companion animals’ strains (*n =* 58)	Humans strains (*n =* 53)	Companion animals’ strains (*n =* 24)	Humans strains (*n =* 5)
	%R (No)	%R (No)	%R (No)	%R (No)
Amikacin	1.7 (1)	0 (0)	0 (0)	0 (0)
Amoxicillin/clavulanate	36.2 (21)	7.5 (4)	79.2 (19)	80 (4)
Ampicillin/sulbactam	84.5 (49)	71.7 (38)	95.8 (23)	100 (5)
Ampicillin	94.8 (55)	100 (53)	87.5 (21)	100 (5)
Aztreonam	81 (47)	60.4 (32)	91.7 (22)	80 (4)
Ceftazidime	77.6 (45)	73.6 (39)	95.8 (23)	100 (5)
Cefepime	75.9 (44)	88.7 (47)	50 (12)	20 (1)
Cefotaxime	100 (58)	100 (53)	100 (24)	100 (5)
Cefoxitin	20.7 (12)	7.5 (4)	70.8 (17)	80 (4)
Cephalothin	100 (58)	100 (53)	100 (24)	100 (5)
Ciprofloxacin	63.8 (37)	43.4 (23)	16.7 (4)	0.2 (1)
Chloramphenicol	37.9 (22)	15.1 (8)	29.2 (7)	0 (0)
Ertapenem	5.2 (3)	1.9 (1)	16.7 (4)	0 (0)
Gentamicin	18.9 (11)	7.6 (4)	29.2 (7)	0 (0)
Imipenem	0 (0)	0 (0)	16.7 (4)	0 (0)
Meropenem	0 (0)	0 (0)	16.7 (4)	0 (0)
Piperacillin/tazobactam	17.2 (10)	7.6 (4)	25 (6)	0 (0)
Trimethoprim/sulfamethoxazole	53.5 (31)	67.9 (36)	37.5 (9)	0 (0)
Multidrug-resistant	86.2 (50)	66 (35)	54.2 (13)	0 (0)

Minimum inhibitory concentrations (MICs) were interpreted using the criteria of the European Committee on Antimicrobial Susceptibility Testing (EUCAST) 2024 (The European Committee on Antimicrobial Susceptibility Testing, 2024), except for amoxicillin/clavulanate, ampicillin/sulbactam, and trimethoprim/sulfamethoxazole, for which criteria from the Clinical and Laboratory Standards Institute (CLSI) were used (CLSI, 2024). Data are reported as a number (No) of resistant (R) strains; *n*, total number of non-duplicate strains tested.

In the United Kingdom, resistance rates for companion animal strains were high for ampicillin/sulbactam (95.8) and aztreonam (91.7%). However, the resistance to ciprofloxacin was lower in companion animal strains (16.7%) compared to Portugal. Human strains showed 100% resistance to ampicillin. None of the human strains presented a multidrug-resistant profile, and only 54.2% of the companion animal strains did (Table 3).

#### 3.2.4 Genes conferring resistance to third-generation cephalosporins and carbapenems

**Figure 1:**
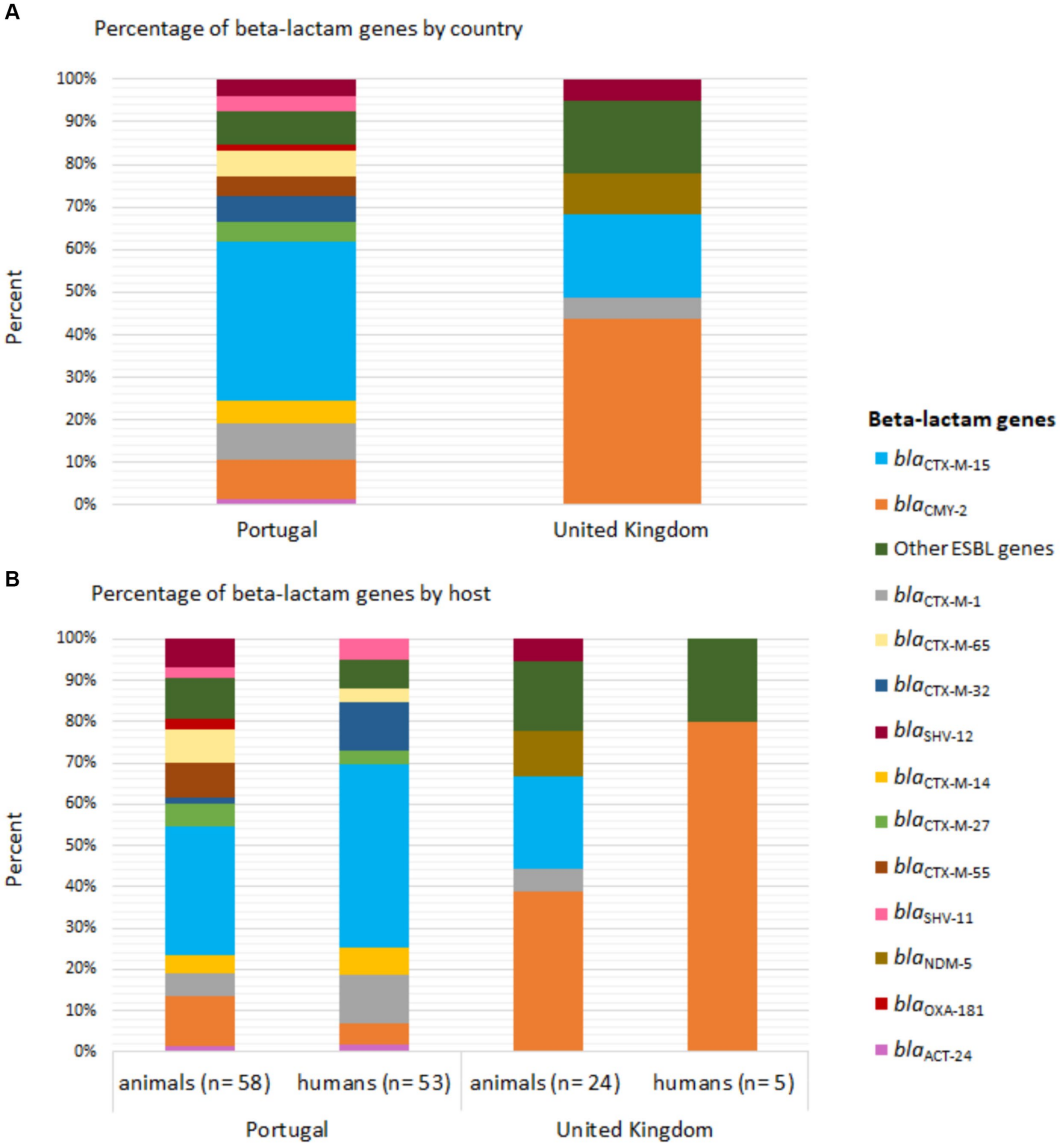
Distribution of beta-lactam genes in Enterobacterales strains carried by companion animals and their cohabitant humans in Portugal and the United Kingdom. **(A)** Percentage of beta-lactam genes by country and **(B)** host. The bars are color-coded according to the gene, as indicated in the legend inset.

The distribution of beta-lactam resistance genes varied between strains from the two countries. The *bla*_CTX-M-15_ gene was the most frequent in strains from Portugal (humans: 49.1%, *n =* 26/53; animals: 39.7%, *n =* 23/58), followed by *bla*_CTX-M-1_ and *bla*_CTX-M-32_ in the humans (13.2%, *n =* 7/53) and *bla*_CMY-2_ in animals (15.5%, *n =* 9/58) (Figure 1). In the UK, the *bla*_CMY-2_ gene was the most common (humans: 80%, *n =* 4/5; animals: 58.3%, *n =* 14/24) (Figure 1).

Furthermore, carbapenemase-encoding genes were found in *E. coli* strains recovered from meropenem and temocillin-supplemented MacConkey agar plates. In Portugal, a dog carried the *bla*_OXA-181_ gene in two meropenem-susceptible *E. coli* strains; in the UK, four meropenem-resistant strains harboring the *bla*_NDM-5_ gene were isolated from one dog, as has been previously reported (Brilhante et al., 2020; Menezes et al., 2023).

### 3.3 Longitudinal isolation of beta-lactam-resistant Enterobacterales carriage strains

**Figure 2:**
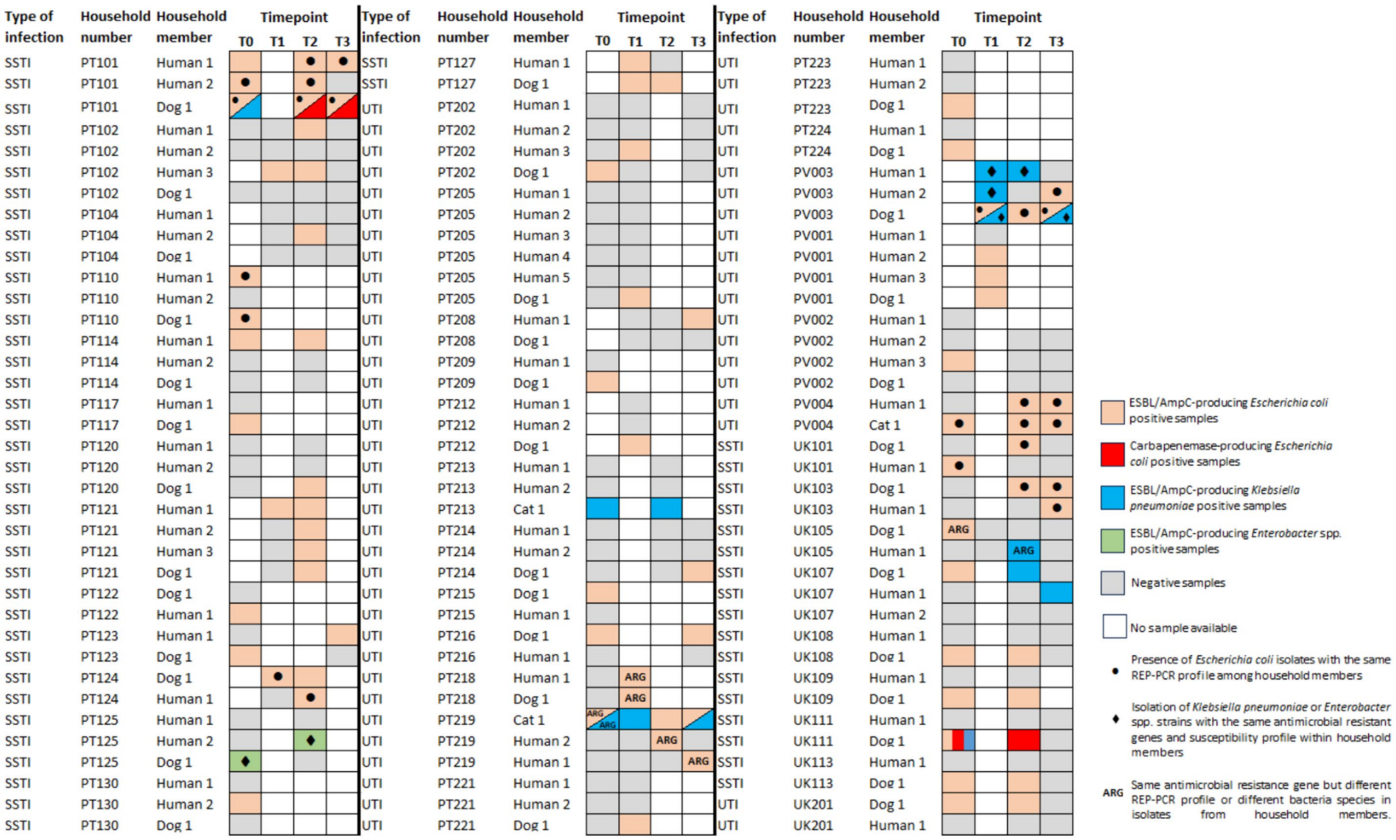
Distribution of ESBL/AmpC- or carbapenemase-producing Enterobacterales across fecal/nasal swabs sampling timepoints of 32 positive households in Portugal (PT) and nine households from the United Kingdom (UK). See the color key on the right side of the figure. SSTI, skin and soft tissue infection; UTI, urinary tract infection.

In Portugal, of the 43 households enrolled, 32 had at least one participant who carried an ESBL/AmpC-E and/or CPE strain. As the collection of follow-up samples was dependent on the owner’s willingness to continue to participate in the study, only 22 of the 32 positive households delivered samples at two or more timepoints (Figure 2). Repeated isolation of ESBL/AmpC-E strains was observed for eight humans and eight companion animals from 11 households (Figure 2). Persistence of the same ESBL/AmpC-producing *E. coli* strains was detected by REP-PCR profiling in three humans (PT101H1, PT101H2, and PV004H1) and four companion animals (PT101D1, PT127D1, PV003D1, and PV004C1) (Figure 2; Supplementary Figure S3).

In the UK, nine of the 22 households had at least one participant colonized by an ESBL/AmpC-E and/or CPE strain. All positive households had follow-up samples (Figure 2). Two dogs had the same *E. coli* strain identified in subsequent samples (UK103D1 and UK111D1) (Supplementary Figure S4); another dog (UK109D1) had *K. pneumoniae* strains with the same susceptibility profile and resistant genes observed in different timepoints (Figure 2).

The acquisition of ESBL/AmpC-E strains by companion animals that were initially negative (PT: *n* = 32; UK: *n* = 15) occurred in five (15.6%) companion animals from Portugal (1 week after treatment for three dogs; 1 month after treatment for another dog; and 2 months after treatment for another dog) and in two dogs from the UK (13.3%), 1 month after treatment (Figure 2).

### 3.4 Bacterial strains causing infection in companion animals

A variety of bacterial strains were diagnosed as causes of infections in the 65 companion animals enrolled. Most of the SSTIs in both countries were caused by staphylococci, while UTIs were mostly due to *E. coli* (Supplementary Table S3). In Portugal, a total of 22 Enterobacterales clinical strains were isolated, of which 10 of them (47.6%) were resistant to 3GCs. Molecular analyses showed the presence of ESBL/AmpC encoding genes in all of them (Table 4). Regarding UK clinical strains, seven strains were identified as Enterobacterales, and only two displayed resistance to 3GCs. These resistant strains were not subjected to further investigation.

**Table 4:** Genotypic characteristics of third- and fourth-generation cephalosporin-resistant Enterobacterales strains causing infection in companion animals (*n* = 10).

Household	Host	Type of infection^a^	Strain code	Bacterial species	Pattern of resistance^b^	Beta-lactam-resistant genes
PT110	Dog	SSTI	PT110/0-D1F3E1	*Escherichia coli*	AMC, AMP, C, CIP, CFL, CTX, F, FOX, TMS	*bla*_CTX-M-65_, *bla*_TEM-1_
PT125	Dog	SSTI	PT125/0-D1I3E1	*Escherichia coli*	AK, AMP, CAZ, CFL, CIP, CTX, CPM, F	*bla*_CTX-M-27_
PT202	Dog	UTI	PT202/0-D1I3E1	*Escherichia coli*	AMP, CIP, CFL, CTX, TMS	*bla*_CTX-M-1_, *bla*_TEM-1_
PT209	Dog	UTI	PT209/0-D1I3E1	*Escherichia coli*	AMC, AMP, CAZ, CFL, CN, CTX, F, FOX, TMS	*bla*_CMY-2_, *bla*_TEM-1_
PT212	Dog	UTI	PT212/0-D1I4E1	*Escherichia coli*	AMP, CAZ, CFL, CIP, CTX, FOX	*bla*_CTX-M-15_
PT213	Cat	UTI	PT213/T0-C1I3K1	*Klebsiella pneumoniae*	AMC, AMP, CAZ, CFL, CN, CPM, CTX, F, FOX, TET, TMS	*bla*_CTX-M-15_, *bla*_SHV-28_, *bla*_TEM-1_
PV001	Dog	UTI	PV001G1D1U1	*Enterobacter* spp.	AMC, AMP, CAZ, CFL, C, CIP, CN, CTX, F, FOX, TET, TMS	*bla*_CMY-2_
PV002	Dog	UTI	PV002V1UD1U1	*Klebsiella pneumoniae*	AMC, CAZ, CIP, CFL, CN, CTX, CPM, TMS	*bla*_CTX-M-15_, *bla*_CMY-2_, *bla*_TEM-1_
PV003	Dog	UTI	PV003V1UD1K5	*Klebsiella pneumoniae*	AMP, CAZ, CFL, CTX, CPM, F, TET, TMS	*bla*_CTX-M-15_, *bla*_SHV-11_, *bla*_TEM-1_
PV004	Cat	UTI	PV004V0C1I0E1	*Escherichia coli*	AMP, CAZ, CFL, CIP, CTX, CPM, TET, TMS	*bla*_CTX-M-27_

^a^ UTI, urinary tract infection; SSTI, skin and soft tissue infection.

^b^ Antibiotics tested: AK, amikacin; AMC, amoxicillin/clavulanate; AMP, ampicillin; C, chloramphenicol; CAZ, ceftazidime; CFL, cephalothin; CIP, ciprofloxacin; CN, gentamicin; CTX, cefotaxime; CPM, cefepime; ETP, ertapenem; F, nitrofurantoin; FOX, cefoxitin; IMP, imipenem; MEM, meropenem; TET, tetracycline; TMS, trimethoprim/sulfamethoxazole.

Minimum inhibitory concentrations (MICs) were interpreted using the criteria of the European Committee on Antimicrobial Susceptibility Testing (EUCAST) 2024 (The European Committee on Antimicrobial Susceptibility Testing, 2024), except for amoxicillin/clavulanate, ampicillin/sulbactam, and trimethoprim/sulfamethoxazole, for which criteria from the Clinical and Laboratory Standards Institute (CLSI) were used (CLSI, 2024).

Among clinical ESBL/AmpC-producing Enterobacterales strains from Portugal, a *K. pneumoniae* (PV003V1UD1K5) and two *E. coli* (PT110/0-D1I3E1 and PV004V0C1I0E1) strains from different animals presented the same genetic profile and resistance pattern as carriage strains from the respective companion animal and their cohabiting humans. To further explore the genetic relatedness of these clinical and carriage strains, one representative carriage strain from each household member per timepoint and the clinical strains were analyzed by WGS. In a total, 9 *E. coli* and 7 *K. pneumoniae* strains were analyzed by WGS.

### 3.5 Co-carriage of ESBL/AmpC-producing Enterobacterales strains

The presence of ESBL/AmpC-E carriage strains in companion animals and cohabitant humans harboring the same resistance genes was observed in eight Portuguese households (18.6%, *n =* 8/43) and two households from the UK (9.1%, *n =* 2/22) (Figure 2). Of these, five households (PT = 3; UK = 2) included companion animal–human *E. coli* paired strains with matching REP-PCR that were selected for WGS analysis (one representative strain from each household member per timepoint, comprising a total of 20 *E. coli* strains).

For another household, *E. hormaechei* strains were recovered from an animal/owner pair (Figure 2) presenting the same resistant genes and susceptibility profile; these strains (*n* = 2) were also sequenced.

For one household from the UK (UK105), the *bla*_CMY-2_ gene was associated with an *E. coli* in the dog and a *K. pneumoniae* in the owner; in a similar way, in a household from Portugal (PT219), the *bla*_CTX-M-15_ gene was found in a *K. pneumoniae* and *E. coli* from the cat and at *E. coli* strains from its cohabitant humans. These strains were not sequenced as companion animals’ and owners’ strains belonged to different bacterial species and different *E. coli* REP-PCR profiles (Figure 2; Supplementary Table S2).

### 3.6 Core genome relatedness between animals and humans’ strains

**Figure 3:**
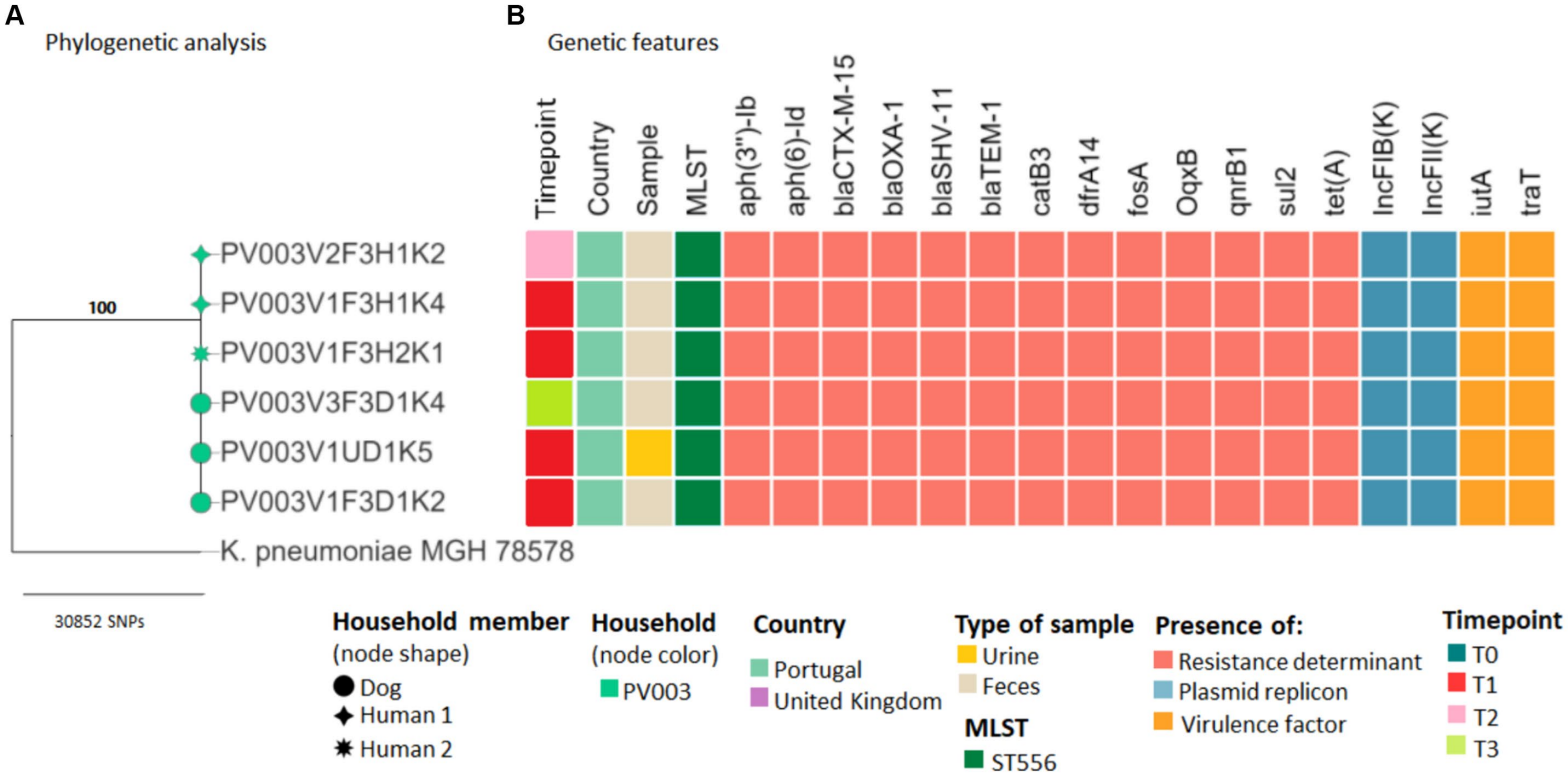
Core genome SNP analysis and genetic features of clinical and carriage ESBL/AmpC-producing *Klebsiella pneumoniae* strains from companion animals and their cohabitant humans. **(A)** Maximum likelihood phylogeny of the core genome of six *K. pneumoniae* strains and the *K. pneumoniae* subsp. *pneumoniae* MGH 78578 strain. Bootstrap support values are shown in bold on each node. **(B)** Heatmap shows the sequence types, antimicrobial resistance determinants, plasmid replicons, and virulence factors for each strain (see color key at the bottom of the figure).

Evidence of sharing was found in several households. In household PV003*, K. pneumoniae* ST556 strains harboring the *bla*_CTX-M-15_ gene, recovered from dog and owners’ fecal samples at different timepoints, had ≤7 SNP differences from animals’ clinical strains, indicating possible transmission of the pathogenic bacteria (Figure 3; Supplementary Table S4).

**Figure 4:**
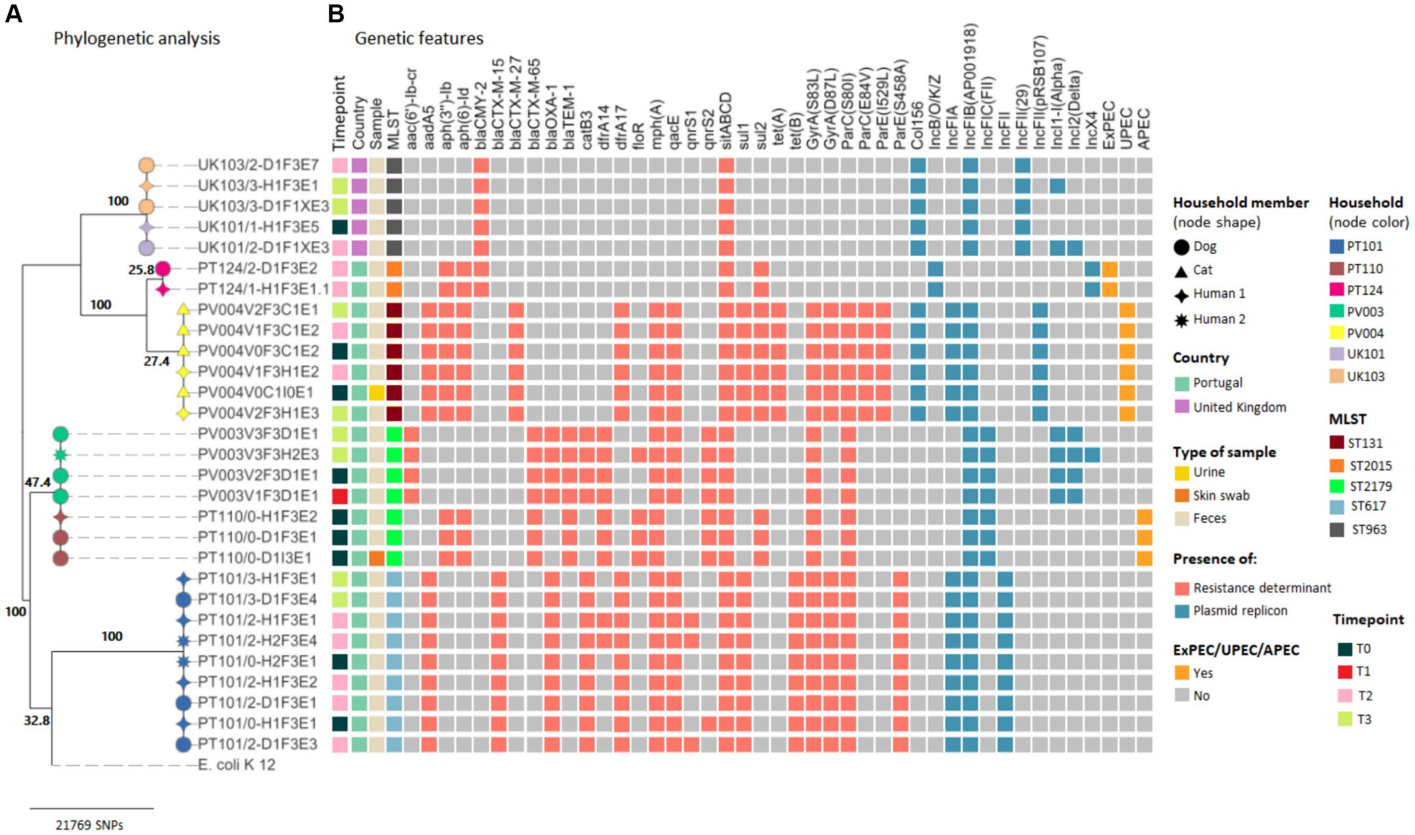
Core genome SNP analysis and genetic features of clinical and carriage ESBL/AmpC-producing *Escherichia coli* strains from companion animals and their cohabitant humans. **(A)** Maximum likelihood phylogeny of the core genome of 33 *E. coli* strains and the *E. coli* K-12 MG1655 strain. Bootstrap support values are shown in bold on each node. **(B)** Heatmap shows the *E. coli* sequence types, antimicrobial resistance determinants, plasmid replicons, and pathotypes based on a repertoire of virulence factors for each strain (see color key on the right side of the figure).

Maximum likelihood phylogenetic tree based on the SNP alignment of 29 *E. coli* core genomes showed six large clusters corresponding to five different households from Portugal and one cluster comprising strains from two UK households (Figure 4).

In two of the Portuguese households clusters (PT110 and PV004), the sharing of companion animal’s *E. coli* clinical strains was confirmed (Figure 4). At household PT110, an *E. coli* ST2179 strain harboring the *bla*_CTX-M-65_ and *bla*_TEM-1_ genes causing SSTI in the dog was shared between the pet and owner (2 SNP difference). These strains were classified as APEC due to the presence of *hlyF, iutA, iroN, iss, and ompT* virulence genes (Supplementary Table 5).

For household PV004, the cat–human pair was colonized by an *E. coli* ST131 strain harboring the *bla*_CMY-2_ gene causing UTI in the cat. Cat’s clinical and colonization strains from T0, T2, and T3, as well as its owner’s strains, recovered at timepoint T2 and T3, displayed ≤3 SNP difference (Figure 3; Supplementary Table S6). The strains from household PV004 displayed virulence genes typically associated with UPEC (*chuA, fyuA, and yfcV*) (Supplementary Table S5).

Among the remaining three Portuguese household clusters (PV003, PT101, and PT124), analysis of the core genomes confirmed that no distinction could be made between *E. coli* carriage strains from the three companion animal–human pairs as within-household strains presented ≤4 SNPs differences (Figure 4). For households, PT101 sharing over time of the *E. coli* ST617 strains harboring *bla*_CTX-M-15_ was also observed as strains were isolated from the dog at T2 and T3 and its owners at T0, T2, and T3. Furthermore, clonal strains presented the same MLST result, the same resistance genes, plasmid replicons, and pathotypes (Figure 4; Supplementary Table S5). Strains from household PT124 displayed virulence genes typically associated with pathogenic *E. coli* strains and were classified as ExPEC (*kpsMII, papC, and sfaD*) (Figure 4; Supplementary Table S5).

The strains from the two UK households clustered together (UK101 and UK103), presented only 9 SNPs of difference between them (Figure 4; Supplementary Table S6). These results demonstrated that sharing of the same ESBL/AmpC-producing *E. coli* carriage strains occurred between companion animals and owners and between participants from different households in the UK. These *E. coli* strains belong to ST963 harboring the *bla*_CMY-2_ gene_._

In addition to the ESBL/AmpC genes, sequenced strains exhibited a wide variety of genes encoding for narrow-spectrum beta lactamases (*bla*_TEM–1_ and *bla*_OXA-1_) and resistance against aminoglycosides [*aadA5*, *aph(6)-Id, and aph(3″)-Ib*], fluoroquinolones (*qnrS and qnrB1*), macrolides [*mph*(A)], phenicols (*catB3 and floR*), sulphonamides (*sul1* and *sul2*), trimethoprim (*dfrA14* and *dfrA17*), tetracyclines [*tet*(A) and *tet*(B)], and point mutations in *gyrA*, *parC*, and/or *parE* genes, which could confer resistance to nalidixic acid and fluoroquinolones (Figures 3, 4; Supplementary Table S5).

**Figure 5:**
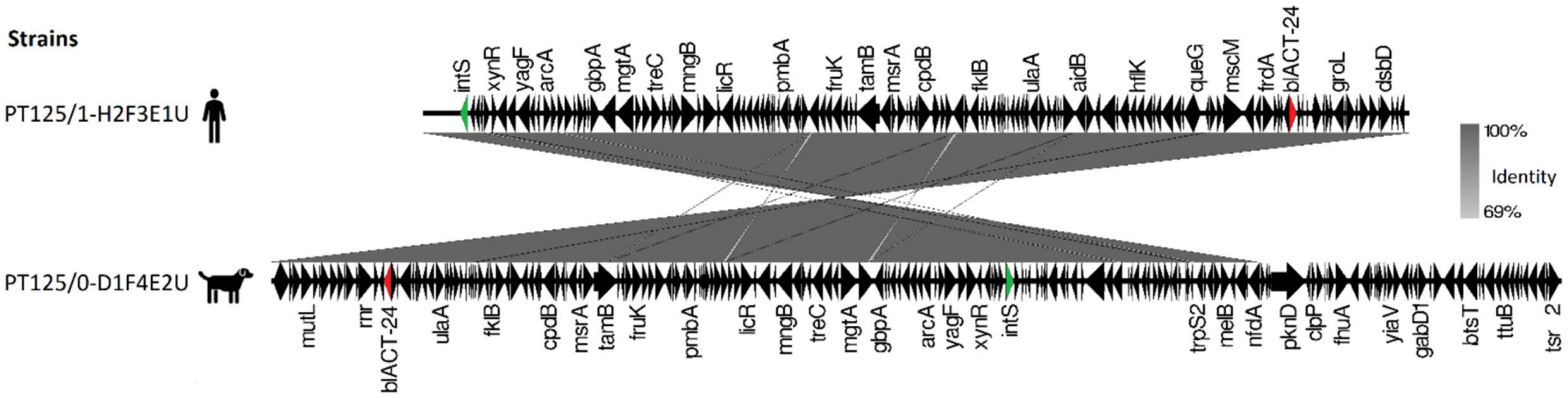
Map of *bla*_ACT-24_ genetic environment comparison between carriage *Enterobacter hormaechei* subsp. *hoffmannii* strains from a dog and their cohabitant human fecal samples. Antibiotic resistance genes are represented by red arrows, integrase by green arrows, and other genes with black arrows.

*E. hormaechei* dog-owner paired strains share high nucleotide similarity presenting 16 SNPs difference (Supplementary Table S7). These strains harbored a *bla*_ACT-24_ gene, flanked by multiple regulon genes and an integrase, suggesting that it has been integrated into the chromosomal DNA (Figure 5).

## 4 Discussion

The close relationship between pets and humans supports the likelihood of transferring antibiotic-resistant bacteria and resistance-mediating mobile genetic elements, between them (Pomba et al., 2017). Here, we presented insights on the co-carriage of ESBL/AmpC-E and CPE strains between companion animals receiving antimicrobials and their owners in Portugal and the UK, as well as the transmission of animals’ clinical strains within the household. WGS combined with phylogenetic analysis was used to infer bacterial resistance transmission dynamics.

This study used a prospective, longitudinal design to examine the effect of antibiotic treatment on the selection and carriage of ESBL/AmpC-E and CPE strains in a cohort of dogs and cats with UTI or SSTI. The prevalence of ESBL/AmpC-E carriage in companion animals under antibiotic therapy in this study (PT = 55.8%; UK = 36.4%) was higher compared to the prevalence in healthy animals in both countries (12.7 and 8.5% for Portugal and the UK, respectively) during the same timeframe (Menezes et al., 2023). While care must be taken when comparing data from different studies, the similarity in recruitment and methods between these studies allows for a meaningful comparison. The critical distinction that one study focused on healthy animals, while the current study involves companion animals under antibiotic therapy, supports the potential for antibiotic selection pressure being exerted upon the animals in the current study based on the observed prevalence rates. Furthermore, in the present study, 15.6% of the companion animals from Portugal and 13.3% from the UK acquired ESBL/AmpC-E strains after antibiotic treatment. These findings suggest a role for companion animals under antibiotic treatment as reservoirs of AMR that may be potentially transmissible to the environment and to other hosts, including their owners.

Despite the absence of significant differences, the prevalence of ESBL/AmpC-E strains in companion animals was higher than in their owners (PT = 35.9%; UK = 12.5%), following the pattern previously found in other studies (Abbas et al., 2019; Dazio et al., 2021) that suggest companion animals as a source of this type of resistance in a household.

The observed differences in the detection of ESBL/AmpC-E strains between Portuguese and British individuals align with the broader patterns of higher prevalence of 3GC-resistant Enterobacterales isolates in Southern European countries compared to Northern European countries (Marques et al., 2016; WHO Regional Office for Europe/European Centre for Disease Prevention and Control, 2022). This suggests that geographical variation could be associated with the acquisition of resistant bacteria, potentially emerging as a risk factor. Such geographical disparities may reflect underlying regional differences in antimicrobial usage. In Portugal, there is a higher rate of antimicrobial use in the community, especially for systemic treatments with cephalosporins and other beta-lactam agents, compared to England (European Centre for Disease Prevention and Control, 2023; UK Health Security Agency, 2023). However, the substantial discrepancy in sample size between Portugal (*n* = 78 humans) and the UK (*n* = 24) might have also contributed to the observed difference. Although statistical adjustments were made to account for these variations, the impact of sample size on the robustness of our comparisons and the generalizability of our findings should be considered.

CPE carriage was only found in companion animals. This finding was somewhat surprising as such isolates are primarily reported in human clinical samples and carbapenems are not authorized for veterinary use (EMA, 2019; World Health Organization, 2024). The lack of positive cases in humans may be attributed to the limited sample size featured in this study. In addition, recent studies have shown that carbapenemase carriage is generally rare among healthy individuals globally (Diebold et al., 2023). Hospital settings, however, are known to harbor antimicrobial resistance genes, making carbapenemase genes more common in inpatient and outpatient cohorts compared to healthy individuals who were the focus of our study. In contrast, the animals in our study were exposed to veterinary hospital environments, where antibiotic use could facilitate the co-selection of resistant strains, potentially explaining their CPE carriage. These findings emphasize the importance of incorporating dogs/cats in the assessment of antimicrobial resistance circulation in the community setting.

We did not identify any risk factors for ESBL/AmpC-E or CPE carriage. In previous studies, raw food diets and prior antimicrobial use have been identified as risk factors for ESBL-E carriage in companion animals (Baede et al., 2017; Schmidt et al., 2018). However, no association between the consumption of raw food and ESBL/AmpC-E or CPE carriage was found (*p* = 0.6817 in Portugal; *p* = 0.7175 in the UK) or between antimicrobial treatment 3–12 months prior to sampling and carriage (*p* = 0.9121 in Portugal and *p =* 0.9333 in the UK). Yet, the acquisition of resistant bacteria was observed after a single course of antibiotics. Similarly, hospital setting contact has also been strongly associated with AMR carriage (Adler et al., 2016; Nigg et al., 2019); here, being a healthcare professional (*p* = 0.9206 in Portugal; *p* = 0.9792 in the UK) or recently hospitalized (≤12 months) (*p* = 0.9680 in Portugal; *p* = 0.9792 in the UK) was not established as risk factors among humans participants. This may be explained by the small sample size of participants presenting these characteristics, which was not powered to detect changes. Although given the extensive dissemination of ESBL/AmpC-E strains, there are numerous routes for acquiring these resistant strains, making the identification of risk factors difficult (Salgado-Caxito et al., 2021; WHO Regional Office for Europe/European Centre for Disease Prevention and Control, 2022; Zamudio et al., 2022).

*E. coli* was the most predominant species identified in 3CG-resistant carriage isolates, suggesting that this bacterial species may act as a reservoir of ESBL/AmpC-E genes. The detection of phylogroup B2 and D strains in high rates of occurrence in the community emphasizes the need for ongoing surveillance. These two phylogroups are commonly associated with strains causing extraintestinal infection, namely, urinary tract infection (Schmiedel et al., 2014).

ESBL/AmpC-producing *K. pneumoniae* was the second most found among carriage strains. *K. pneumoniae* stands as a healthcare-associated pathogen known for causing various infections and its ability to develop antibiotic resistance (WHO Regional Office for Europe/European Centre for Disease Prevention and Control, 2022). The prevalence reported here for ESBL/AmpC-producing *K. pneumoniae* is higher in contrast with previous observations by our group in healthy companion animals and humans in both countries (Menezes et al., 2023) but in agreement with studies in dogs and cats presented to veterinary hospitals in Switzerland (12.3%) (Dazio et al., 2021). Also of notice is the high proportion of multidrug-resistant ESBL/AmpC-E strains pointing toward a rising trend of AMR within the community setting.

Despite the ubiquitous nature of resistance genes, there was a variation in their distribution and frequency between geographical origins. The *bla*_CTX-M-15_ gene was more frequent in Portugal compared to the UK, where the *bla*_CMY-2_ gene was more common. These observations are consistent with previous resistance determinant patterns observed in both countries (Wedley et al., 2017; Carvalho et al., 2021). On the other hand, the *bla*_ACT-24_ gene was only identified in shared *E. hormaechei* strains. *Enterobacter* spp. strains often harbor an inducible chromosomal *ampC* gene encoding AmpC beta-lactamase (Meini et al., 2019). The *bla*_ACT-24_ gene has been identified before as chromosomally integrated in *E. hormaechei* strains (Lasarte-Monterrubio et al., 2023). In our study, this resistant gene was found surrounded by an integrase indicating that it was chromosomally integrated and highlighting its potential for mobilization and spread within microbial communities.

Transmission of bacteria strains causing infections in companion animals to their household members has been sporadically reported, encompassing bacteria resistant to medically important antimicrobials, such as NDM-5-producing *E. coli* (Grönthal et al., 2018), as well as dog-derived UTI ExPEC strains resulting in UTI in the owners (Johnson et al., 2008), and the reverse scenario has also been described (Johnson and Clabots, 2006; Johnson et al., 2008; Toombs-Ruane et al., 2020). In the present study, three households from Portugal had owners carrying the ESBL/AmpC-E strains causing infection in their cohabiting companion animal. The shared index *E. coli* strains belong to UPEC and APEC pathotypes. UPEC is the primary cause of urinary tract infections in humans, while APEC typically causes a range of infections in poultry (Geurtsen et al., 2022). Intriguingly, studies have shown that APEC exhibits genetic similarity with human ExPECs, including the possession of virulence genes associated with UTIs and meningitis (Tivendale et al., 2010; Mellata, 2013), thus emphasizing its potential for zoonotic transmission. The co-occurrence of both variants causing infection in companion animals and their owners’ fecal samples is particularly noteworthy, indicating that companion animals may act as reservoirs and disseminators of these strains. This highlights the importance of infection control measures during animal disease.

Overall, here we documented the sharing of carriage of ESBL/AmpC-E between companion animals and owners within the same households in Portugal (13.9%, *n* = 6/43) and the UK (9.1%, *n* = 2/22) based on core genome analysis. When considering only households with positive participants for ESBL/AmpC carriage, the sharing frequency from Portugal increases to 18.8% (*n* = 6/32) and 22.2% in the UK (*n* = 2/9). These frequencies are higher compared to the ones found among households with healthy companion animals in both countries (sharing of 4.9% in Portugal and 0% in the UK) (Menezes et al., 2023). These differences could be associated with the animal’s exposure to antibiotic selective pressure, as mentioned above, that increased carriage, making the occurrence of sharing more likely. Nevertheless, in a similar study to ours, no co-carriage was observed between dogs and cats presented to veterinary clinics and hospitals in Switzerland and their owners (Dazio et al., 2021). This could be related to the unique patterns of antimicrobial usage and resistance observed in both animals and humans across different geographical locations, as reported elsewhere (European Centre for Disease Prevention and Control, 2023; European Medicines Agency and European Surveillance of Veterinary Antimicrobial Consumption, 2023; UK Health Security Agency, 2023). Southern European countries, such as Portugal, generally exhibit higher levels of antimicrobial resistance compared to Northern countries (Marques et al., 2016; WHO Regional Office for Europe/European Centre for Disease Prevention and Control, 2022). Consequently, places with higher resistance levels have an increased likelihood of sharing resistant bacteria.

Although a high prevalence of intra-familial co-carriage was observed, underestimation cannot be excluded since data rely on bacterial culture, a methodology that has a low sensitivity (Tofteland et al., 2007).

Shared strains within households belonged to five different *E. coli* sequence types (ST131, ST2015, ST2179, ST617, and ST963), *K. pneumoniae* ST556, and *E. hormaechei,* which include bacterial lineages that have been already reported in the animal and human settings before (Jure et al., 2019; Sepp et al., 2019; Salgado-Caxito et al., 2021; Donà et al., 2023). Although direct transmission cannot be confirmed, the diminutive number of SNP differences suggests very recent acquisition from a common source or recent direct transmission. The last one could have occurred via petting, cuddling, licking the owner’s face, or cleaning the pet’s waste, behaviors that were reported on the questionnaires to occur at least occasionally in over 90% of the households. Determining the direction of transmission or whether both humans and dogs were contaminated by the same source is challenging, given that the study design precluded its assessment. Numerous potential routes of exposure exist, including common food and water, the surrounding household environment, and contaminated hospital environment.

In two households from the UK (UK101 and UK103), dogs and their cohabitant humans carried indistinguishable *E. coli* ST963 strains, indicating a common source of exposure. Notably, both dogs attended the same veterinary hospital, where they may have acquired the bacteria through a contaminated hospital environment and then transmitted it to their owners. Veterinary hospitals have been implicated in the selection and dissemination of AMR among patients (Nigg et al., 2019; Dazio et al., 2021). The lack of rigorous infection prevention and control (IPC) measures in these settings exacerbates this issue (Willemsen et al., 2019). Implementing robust IPC programs tailored to veterinary clinics is crucial to mitigate the occurrence of transmission events that could further spread to the animal and human population and the environment.

Further exhaustive study into plasmids harboring antimicrobial resistance, coupled with the role of other mobile genetic elements, including transposons, in the transmission and epidemiology of these resistance genes, is needed. This constitutes a significant limitation of the current study as there is the potential for plasmid-mediated gene transfer to occur, for example, as in the case of the *bla*_CMY-2_ and *bla*_CTX-M-15_ genes, which were associated with two different bacterial species in the UK105 and PT219 households, respectively. Another limitation is the noteworthy proportion of participants failing to conclude the longitudinal study, notwithstanding favorable initial recruitment. Consequently, this study may potentially underestimate the prevalence of persistent carriage of ESBL/AmpC-E and CPE strains.

This study highlights the need for long-term, globally standardized monitoring of AMR among companion animals and cohabiting humans to understand the dynamics and relative significance of the various sources influencing the burden of AMR over time. The potential for inadvertent selection of antibiotic-resistant colonizing bacteria should be considered in prospective antimicrobial stewardship strategies aimed at limiting the development and dissemination of AMR and thus the importance of adopting a One Health approach that integrates both human and animal health.

## Data Availability

The datasets presented in this study can be found in online repositories. The names of the repository/repositories and accession number(s) can be found at: https://www.ebi.ac.uk/ena, PRJEB55525. High resolution *E. coli* phylogenetic tree linked to molecular data is available at Microreact platform, https://microreact.org/project/6r3o1DHTVwF5qPVK4LbPpM-e-coli-transmission-during-different-types-of-animal-infection; *K. pneumoniae* phylogenetic tree can also be found at https://microreact.org/project/iHYMJ8K9dZpkzctDeCprdd-klebsiella-pneumoniae-sharing.
