# Supplementary material for: Transmission dynamics of ESBL/AmpC and carbapenemase-producing Enterobacterales between companion animals and humans
Source: Front Microbiol. 2024 Sep 3;15:1432240. doi: 10.3389/fmicb.2024.1432240 (PMC11405340; doi:10.3389/fmicb.2024.1432240)
Supplement: Supplementary file 1 [file Image_1.pdf]

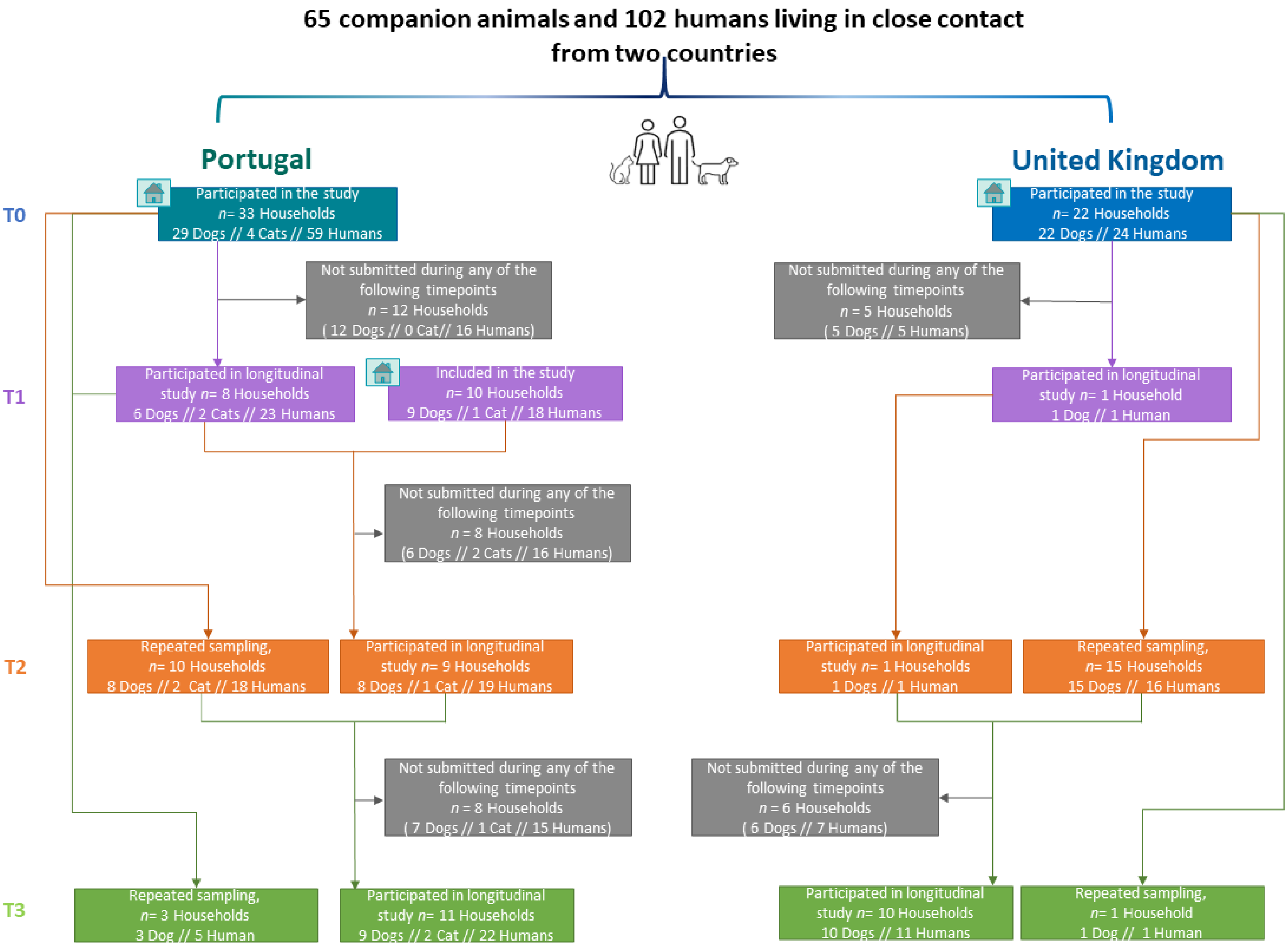

65 companion animals and 102 humans living in close contact from two countries
Portugal
United Kingdom
T0
T1
T2
T3
Participated in the study n= 33 Households 29 Dogs // 4 Cats // 59 Humans
Not submitted during any of the following timepoints n = 12 Households (12 Dogs // 0 Cat// 16 Humans)
Participated in longitudinal study n= 8 Households 6 Dogs // 2 Cats // 23 Humans
Included in the study n= 10 Households 9 Dogs // 1 Cat // 18 Humans
Not submitted during any of the following timepoints n = 8 Households (6 Dogs // 2 Cats // 16 Humans)
Repeated sampling, n= 10 Households 8 Dogs // 2 Cat // 18 Humans
Participated in longitudinal study n= 9 Households 8 Dogs // 1 Cat // 19 Humans
Not submitted during any of the following timepoints n = 8 Households (7 Dogs // 1 Cat // 15 Humans)
Repeated sampling, n= 3 Households 3 Dog // 5 Human
Participated in longitudinal study n= 11 Households 9 Dogs // 2 Cat // 22 Humans
Participated in the study n= 22 Households 22 Dogs // 24 Humans
Not submitted during any of the following timepoints n = 5 Households (5 Dogs // 5 Humans)
Participated in longitudinal study n= 1 Household 1 Dog // 1 Human
Participated in longitudinal study n= 1 Households 1 Dogs // 1 Human
Repeated sampling, n= 15 Households 15 Dogs // 16 Humans
Not submitted during any of the following timepoints n = 6 Households (6 Dogs // 7 Humans)
Participated in longitudinal study n= 10 Households 10 Dog // 11 Humans
Repeated sampling, n= 1 Household 1 Dog // 1 Human

Supplementary Figure S1. Flow chart of households' participants by country. House symbol represents insertions of new households to the study. T0 concerns sampling before antimicrobial intake; T1 was done one week after antimicrobial treatment started; T2 one month after antimicrobial treatment started; T3 was achieved 2 months after antimicrobial treatment started; In total, 43 households from Portugal (covering 38 dogs, 5 cats and 78 humans) and 22 households the United Kingdom (covering 22 dogs, and 24 humans) were studied.
