# Supplementary material for: Transmission dynamics of ESBL/AmpC and carbapenemase-producing Enterobacterales between companion animals and humans
Source: Front Microbiol. 2024 Sep 3;15:1432240. doi: 10.3389/fmicb.2024.1432240 (PMC11405340; doi:10.3389/fmicb.2024.1432240)
Supplement: Supplementary file 2 [file Image_2.pdf]

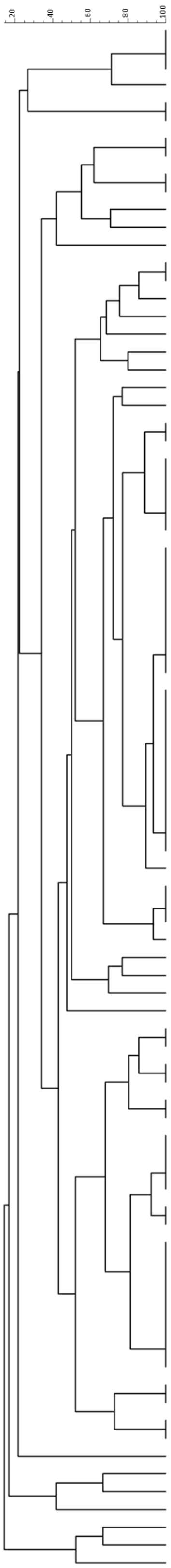

Strain code	Household member	Timepoint	Phylogroup	Beta-lactamase encoding genes $\mathit{bla}_{\text{CTX-M}}$	$\mathit{bla}_{\text{TEM}}$	$\mathit{bla}_{\text{SHV}}$	$\mathit{bla}_{\text{CMY}}$	Carbapenemase
PT124/1-D1F3E1.1	Dog D1	T2	B2	-	-	-	$\mathit{bla}_{\text{CMY-2}}$	-
PT124/2-D1F3E2 ★ (blue)	Dog D1	T2	B2	-	-	-	$\mathit{bla}_{\text{CMY-2}}$	-
PT124/1-H1F3E1.1 ★ (blue)	Human H1	T2	B2	-	-	-	$\mathit{bla}_{\text{CMY-2}}$	-
PT124/2-D1F3E4	Dog D1	T2	B2	-	-	-	$\mathit{bla}_{\text{CMY-2}}$	-
PT121/2-D1F4E1	Dog D1	T2	D	-	-	-	$\mathit{bla}_{\text{CMY-2}}$	-
PT121/2-D1F3E1	Dog D1	T2	D	-	-	-	$\mathit{bla}_{\text{CMY-2}}$	-
PT102/1-H3F3E1	Human H3	T1	B2	$\mathit{bla}_{\text{CTX-M-1}}$	-	-	-	-
PT102/1-H3F3E2	Human H3	T1	B2	$\mathit{bla}_{\text{CTX-M-1}}$	-	-	-	-
PT102/2-H3F3E2	Human H3	T2	B2	$\mathit{bla}_{\text{CTX-M-1}}$	-	-	-	-
PT102/2-H3F3E3	Human H3	T2	B2	$\mathit{bla}_{\text{CTX-M-1}}$	-	-	-	-
PT102/1-H3F3E3	Human H3	T1	B2	$\mathit{bla}_{\text{CTX-M-1}}$	-	-	-	-
PT102/1-H3F3E4	Human H3	T1	B2	$\mathit{bla}_{\text{CTX-M-1}}$	-	-	-	-
PT127/1-H1F3E1	Human H1	T1	B2	$\mathit{bla}_{\text{CTX-M-32}}$	$\mathit{bla}_{\text{TEM-1}}$	-	-	-
PT114/1-H1F3E2	Human H1	T2	A	$\mathit{bla}_{\text{CTX-M-32}}$	-	-	$\mathit{bla}_{\text{CMY-2}}$	-
PT114/1-H1F3E3	Human H1	T2	A	$\mathit{bla}_{\text{CTX-M-32}}$	-	-	$\mathit{bla}_{\text{CMY-2}}$	-
PT117/0-D1F3E1	Dog D1	T0	B1	-	-	$\mathit{bla}_{\text{SHV-12}}$	-	-
PT114/0-H1F3E4	Human H1	T0	A	$\mathit{bla}_{\text{CTX-M-15}}$	-	-	$\mathit{bla}_{\text{CMY-2}}$	-
PT114/1-H1F3E1	Human H1	T2	A	$\mathit{bla}_{\text{CTX-M-32}}$	-	-	-	-
PT102/2-H1F3E1	Human H1	T2	A	$\mathit{bla}_{\text{CTX-M-15}}$	$\mathit{bla}_{\text{TEM-206}}$	-	-	-
PT123/2-H1F3E1	Human H1	T3	A	$\mathit{bla}_{\text{CTX-M-32}}$	-	-	-	-
PT121/2-H1F3E1	Human H1	T2	D	$\mathit{bla}_{\text{CTX-M-32}}$	-	-	-	-
PT102/2-H3F3E1	Human H3	T2	B2	$\mathit{bla}_{\text{CTX-M-1}}$	-	-	-	-
PT101/0-H2F3E2	Human H2	T0	A	$\mathit{bla}_{\text{CTX-M-15}}$	-	-	-	-
PT101/0-H2F3E4	Human H2	T0	A	$\mathit{bla}_{\text{CTX-M-15}}$	-	-	-	-
PT101/2-D1F3E4	Dog D1	T2	A	$\mathit{bla}_{\text{CTX-M-15}}$	-	-	-	-
PT101/2-H1F3E1 ★ (blue)	Human H1	T2	A	$\mathit{bla}_{\text{CTX-M-15}}$	-	-	-	-
PT101/2-D1F3E2	Dog D1	T2	A	$\mathit{bla}_{\text{CTX-M-15}}$	-	-	-	-
PT101/2-D1F3E5	Dog D1	T2	A	$\mathit{bla}_{\text{CTX-M-15}}$	-	-	-	-
PT101/2-D1F3E3 ★ (blue)	Dog D1	T2	A	$\mathit{bla}_{\text{CTX-M-15}}$	-	-	-	-
PT101/0-H1F3E2	Human H1	T0	A	$\mathit{bla}_{\text{CTX-M-15}}$	-	-	-	-
PT101/0-H2F3E3	Human H2	T0	A	$\mathit{bla}_{\text{CTX-M-15}}$	-	-	-	-
PT101/0-H2F3E1 ★ (blue)	Human H2	T0	A	$\mathit{bla}_{\text{CTX-M-15}}$	-	-	-	-
PT101/3-H1F3E1 ★ (blue)	Human H1	T3	A	$\mathit{bla}_{\text{CTX-M-15}}$	-	-	-	-
PT101/3-H1F3E2	Human H1	T3	A	$\mathit{bla}_{\text{CTX-M-15}}$	-	-	-	-
PT101/3-H1F3E3	Human H1	T3	A	$\mathit{bla}_{\text{CTX-M-15}}$	-	-	-	-
PT101/3-H1F3E4	Human H1	T3	A	$\mathit{bla}_{\text{CTX-M-15}}$	-	-	-	-
PT101/3-D1F3E4 ★ (blue)	Dog D1	T3	A	$\mathit{bla}_{\text{CTX-M-15}}$	-	-	-	-
PT101/2-H1F3E2 ★ (blue)	Human H1	T2	A	$\mathit{bla}_{\text{CTX-M-15}}$	-	-	-	-
PT101/2-H1F3E3	Human H1	T2	A	$\mathit{bla}_{\text{CTX-M-15}}$	-	-	-	-
PT101/2-H2F3E4 ★ (blue)	Human H2	T2	A	$\mathit{bla}_{\text{CTX-M-15}}$	-	-	-	-
PT101/0-H1F3E1 ★ (blue)	Human H1	T0	A	$\mathit{bla}_{\text{CTX-M-15}}$	-	-	-	-
PT101/2-D1F3E1 ★ (blue)	Dog D1	T2	A	$\mathit{bla}_{\text{CTX-M-15}}$	-	-	-	-
PT101/2-H2F3E3	Human H2	T2	A	$\mathit{bla}_{\text{CTX-M-15}}$	-	-	-	-
PT101/2-H2F3E1	Human H2	T2	A	$\mathit{bla}_{\text{CTX-M-15}}$	-	-	-	-
PT101/3-D1F3E1	Dog D1	T3	A	$\mathit{bla}_{\text{CTX-M-15}}$	-	-	-	-
PT101/3-D1F3E2	Dog D1	T3	A	$\mathit{bla}_{\text{CTX-M-15}}$	-	-	-	-
PT101/3-D1F3E3	Dog D1	T3	A	$\mathit{bla}_{\text{CTX-M-15}}$	-	-	-	-
PT101/2-H2F3E2	Human H2	T2	A	$\mathit{bla}_{\text{CTX-M-15}}$	-	-	-	-
PT124/1-D1F3E1	Dog D1	T1	B2	$\mathit{bla}_{\text{CTX-M-55}}$	-	-	-	-
PT124/1-D1F3E4	Dog D1	T1	B2	$\mathit{bla}_{\text{CTX-M-55}}$	-	-	-	-
PT124/1-D1F3E6	Dog D1	T1	B2	$\mathit{bla}_{\text{CTX-M-55}}$	-	-	-	-
PT124/1-D1F3E5	Dog D1	T1	B2	$\mathit{bla}_{\text{CTX-M-55}}$	-	-	-	-
PT121/0-H1F3E1	Human H1	T1	D	$\mathit{bla}_{\text{CTX-M-32}}$	-	-	-	-
PT123/0-D1F3E1	Dog D1	T0	D	$\mathit{bla}_{\text{CTX-M-55}}$	$\mathit{bla}_{\text{TEM-135}}$	-	-	-
PT114/0-H1F3E1	Human H1	T0	A	$\mathit{bla}_{\text{CTX-M-15}}$	-	-	-	-
PT123/0-D1F4E1	Dog D1	T0	D	$\mathit{bla}_{\text{CTX-M-55}}$	$\mathit{bla}_{\text{TEM-135}}$	$\mathit{bla}_{\text{SHV-12}}$	-	-
PT127/2-D1F3E1	Dog D1	T2	B	$\mathit{bla}_{\text{CTX-M-1}}$	-	-	-	-
PT127/1-D1F3E1	Dog D1	T2	B	$\mathit{bla}_{\text{CTX-M-1}}$	-	-	-	-
PT101/0-D1F3E4	Dog D1	T0	B	$\mathit{bla}_{\text{CTX-M-65}}$	-	-	-	-
PT101/0-D1F3E3	Dog D1	T0	B	$\mathit{bla}_{\text{CTX-M-65}}$	-	-	-	-
PT121/2-H3F3E1	Human H3	T2	B	$\mathit{bla}_{\text{CTX-M-14}}$	$\mathit{bla}_{\text{TEM-1}}$	-	-	-
PT121/2-H2F3E1	Human H2	T2	B	$\mathit{bla}_{\text{CTX-M-14}}$	$\mathit{bla}_{\text{TEM-1}}$	-	-	-
PT109 ★ (orange)	Dog D1	T2	A	$\mathit{bla}_{\text{CTX-M-15}}$	-	-	-	$\mathit{bla}_{\text{OXA-181}}$
PT101/2-D1F4E2	Dog D1	T2	A	$\mathit{bla}_{\text{CTX-M-15}}$	-	-	-	$\mathit{bla}_{\text{OXA-181}}$
PT101/2-D1F4E3	Dog D1	T2	A	$\mathit{bla}_{\text{CTX-M-15}}$	-	-	-	$\mathit{bla}_{\text{OXA-181}}$
PT101/2-D1F4E4	Dog D1	T2	A	$\mathit{bla}_{\text{CTX-M-15}}$	-	-	-	$\mathit{bla}_{\text{OXA-181}}$
PT101/0-D1F4E1	Dog D1	T0	A	$\mathit{bla}_{\text{CTX-M-15}}$	-	-	-	$\mathit{bla}_{\text{OXA-181}}$
PT113 ★ (orange)	Dog D1	T0	A	$\mathit{bla}_{\text{CTX-M-15}}$	-	-	-	$\mathit{bla}_{\text{OXA-181}}$
PT110/0-H1F3E1	Human H1	T0	B	$\mathit{bla}_{\text{CTX-M-65}}$	$\mathit{bla}_{\text{TEM-1}}$	-	-	-
PT110/0-H1F3E2 ★ (blue)	Human H1	T0	B	$\mathit{bla}_{\text{CTX-M-65}}$	$\mathit{bla}_{\text{TEM-1}}$	-	-	-
PT110/0-H1F3E3	Human H1	T0	B	$\mathit{bla}_{\text{CTX-M-65}}$	$\mathit{bla}_{\text{TEM-1}}$	-	-	-
PT110/0-H1F3E4	Human H1	T0	B	$\mathit{bla}_{\text{CTX-M-65}}$	$\mathit{bla}_{\text{TEM-1}}$	-	-	-
PT110/0-D1F3E1 ★ (blue)	Dog D1	T0	B	$\mathit{bla}_{\text{CTX-M-65}}$	$\mathit{bla}_{\text{TEM-1}}$	-	-	-
PT110/0-D1F3E4	Dog D1	T0	B	$\mathit{bla}_{\text{CTX-M-65}}$	$\mathit{bla}_{\text{TEM-1}}$	-	-	-
PT110/0-D1F3E2	Dog D1	T0	B	$\mathit{bla}_{\text{CTX-M-65}}$	$\mathit{bla}_{\text{TEM-1}}$	-	-	-
PT110/0-D1F3E3	Dog D1	T0	B	$\mathit{bla}_{\text{CTX-M-65}}$	$\mathit{bla}_{\text{TEM-1}}$	-	-	-
PT122/0-H1F3E2	Human H1	T0	B	$\mathit{bla}_{\text{CTX-M-15}}$	$\mathit{bla}_{\text{TEM-135}}$	-	-	-
PT122/0-H1F3E3	Human H1	T0	B	$\mathit{bla}_{\text{CTX-M-15}}$	$\mathit{bla}_{\text{TEM-135}}$	-	-	-
PT124/2-D1F3E5	Dog D1	T2	B	-	$\mathit{bla}_{\text{TEM-1}}$	-	$\mathit{bla}_{\text{CMY-2}}$	-
PT124/2-D1F3E6	Dog D1	T2	B	-	$\mathit{bla}_{\text{TEM-1}}$	-	$\mathit{bla}_{\text{CMY-2}}$	-
PT104/1-H2F3E1	Human H2	T2	A	$\mathit{bla}_{\text{CTX-M-32}}$	-	-	-	-
PT114/0-H1F3E2	Human H1	T0	A	$\mathit{bla}_{\text{CTX-M-15}}$	-	-	-	-
PT124/1-D1F3E7	Dog D1	T1	B	$\mathit{bla}_{\text{CTX-M-55}}$	-	-	-	-
PT130/0-H2F3E3	Human H2	T0	A	$\mathit{bla}_{\text{CTX-M-15}}$	$\mathit{bla}_{\text{TEM-1}}$	-	-	-
PT130/0-H2F3E1	Human H2	T0	A	$\mathit{bla}_{\text{CTX-M-15}}$	$\mathit{bla}_{\text{TEM-1}}$	-	-	-
PT120/1-D1F3E1	Dog D1	T2	B	$\mathit{bla}_{\text{CTX-M-15}}$	$\mathit{bla}_{\text{TEM-206}}$	-	-	-
PT130/0-H2F3E2	Human H2	T0	A	$\mathit{bla}_{\text{CTX-M-15}}$	$\mathit{bla}_{\text{TEM-1}}$	-	-	-

Supplementary Figure S2. Dendrogram based on REP-PCR finger-printing data of 87 *Escherichia coli* carriage strains from companion animals with skin and soft tissue infection and their cohabiting humans from Portugal. Image generated by Bionumerics (Applied Maths, Sint-Martens-Latem, Belgium) software. The first five algorithms on the strains' identification represents the household code number. Blue star represents strains selected for WGS; orange stars identified OXA-181 producing strains from household PT101 previously described by our group (Brilhante *et al.*, 2020); '-', negative for the gene.
