# Supplementary material for: Transmission dynamics of ESBL/AmpC and carbapenemase-producing Enterobacterales between companion animals and humans
Source: Front Microbiol. 2024 Sep 3;15:1432240. doi: 10.3389/fmicb.2024.1432240 (PMC11405340; doi:10.3389/fmicb.2024.1432240)
Supplement: Supplementary file 3 [file Image_3.pdf]

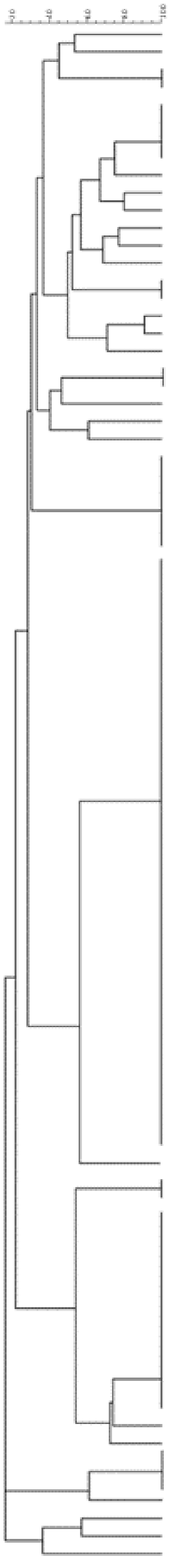

Strain code	Household member	Timepoint	Phylogroup	Beta-lactamase encoding genes: bla_{CTX-M}	bla_{TEM}	bla_{SHV}	bla_{CMY}
PT205/1 D1F3E1	Dog 1	T1	B1	$bla_{CTX-M-1}$	$bla_{TEM-122}$	-	-
PT202/1 H3F3E2	Human H3	T1	A	$bla_{CTX-M-9}$	-	-	-
PT208/3 H1F3E1	Human H1	T3	D	$bla_{CTX-M-15}$	bla_{TEM-1}	-	-
PT208/3 H1F3E2	Human H1	T3	D	$bla_{CTX-M-15}$	bla_{TEM-1}	-	-
PT202/0 D1F3E1	Dog D1	T0	A	$bla_{CTX-M-27}$	-	-	-
PT202/0 D1F3E2	Dog D1	T0	A	$bla_{CTX-M-27}$	-	-	-
PT202/0 D1F3E3	Dog D1	T0	A	$bla_{CTX-M-27}$	-	-	-
PT202/0 D1F3E4	Dog D1	T0	A	$bla_{CTX-M-27}$	-	-	-
PT209/0 D1F3E1	Dog D1	T0	B2	$bla_{CTX-M-14}$	-	-	bla_{CMY-2}
PT214/3 D1F3E1	Dog D1	T3	B1	$bla_{CTX-M-65}$	bla_{TEM-1}	-	-
PT218/1 D1F3E1	Dog D1	T1	B1	$bla_{CTX-M-1}$	-	-	-
PT219/0 C1F3E1	Cat C1	T0	A	-	bla_{TEM-1}	bla_{SHV-12}	-
PT219/2 H2F3E1	Human H2	T2	A	$bla_{CTX-M-15}$	-	-	-
PT218/1 H1F3E1	Human H1	T1	B1	$bla_{CTX-M-1}$	bla_{TEM-1}	-	-
PT221/1 H2F3E1	Human H2	T1	A	$bla_{CTX-M-14}$	-	bla_{SHV-26}	-
PT221/1 H2F3E3	Human H2	T1	A	$bla_{CTX-M-14}$	-	bla_{SHV-26}	-
PT216/0 D1F3E1	Dog D1	T0	B1	$bla_{CTX-M-15}$	-	-	-
PT219/2 C1F3E1	Cat C1	T2	A	-	bla_{TEM-1}	bla_{SHV-12}	-
PT215/0 D1F3E1	Dog D1	T0	A	$bla_{CTX-M-15}$	-	-	-
PT202/1 H3F3E1	Human H3	T1	A	$bla_{CTX-M-9}$	-	-	-
PT202/1 H3F3E3	Human H3	T1	A	$bla_{CTX-M-9}$	-	-	-
PT216/3 D1F3E1	Dog D1	T3	B1	$bla_{CTX-M-15}$	-	-	-
PT215/0 D1F4E1	Dog D1	T0	A	$bla_{CTX-M-15}$	-	-	-
PV002V0F3H3E1	Human H3	T1	A	-	-	-	bla_{CMY-2}
PV001V1F3H2E2	Human H2	T1	A	$bla_{CTX-M-15}$	-	-	-
PV001V1F3H2E4	Human H2	T1	A	$bla_{CTX-M-15}$	-	-	-
PV001V1F3H3E2	Human H3	T1	A	$bla_{CTX-M-15}$	-	-	-
PV001V1F3H3E3	Human H3	T1	A	$bla_{CTX-M-15}$	-	-	-
PV001V1F3H3E4	Human H3	T1	A	$bla_{CTX-M-15}$	-	-	-
PV001V1F3H2E1	Human H2	T1	A	$bla_{CTX-M-15}$	-	-	-
PV004V2F4H1E1	Human H1	T3	B2	$bla_{CTX-M-27}$	-	-	-
PV004V2F4H1E5	Human H1	T3	B2	$bla_{CTX-M-27}$	-	-	-
PV004V2F3H1E3 ★	Human H1	T3	B2	$bla_{CTX-M-27}$	-	-	-
PV004V2F3H1E1	Human H1	T3	B2	$bla_{CTX-M-27}$	-	-	-
PV004V1F4C1E4	Cat C1	T2	B2	$bla_{CTX-M-27}$	-	-	-
PV004V1F3C1E4	Cat C1	T2	B2	$bla_{CTX-M-27}$	-	-	-
PV004V1F4C1E5	Cat C1	T2	B2	$bla_{CTX-M-27}$	-	-	-
PV004V1F3H1E4	Human H1	T2	B2	$bla_{CTX-M-27}$	-	-	-
PV004V1F3H1E5	Human H1	T2	B2	$bla_{CTX-M-27}$	-	-	-
PV004V1F4H1E4	Human H1	T2	B2	$bla_{CTX-M-27}$	-	-	-
PV004V1F4H1E3	Human H1	T2	B2	$bla_{CTX-M-27}$	-	-	-
PV004V2F4C1E6	Cat C1	T3	B2	$bla_{CTX-M-27}$	-	-	-
PV004V2F3C1E5	Cat C1	T3	B2	$bla_{CTX-M-27}$	-	-	-
PV004V2F3C1E3	Cat C1	T3	B2	$bla_{CTX-M-27}$	-	-	-
PV004V2F3C1E2	Cat C1	T3	B2	$bla_{CTX-M-27}$	-	-	-
PV004V2F4C1E6	Cat C1	T3	B2	$bla_{CTX-M-27}$	-	-	-
PV004V1F4H1E2	Humana H1	T2	B2	$bla_{CTX-M-27}$	-	-	-
PV004V1F3C1E2 ★	Cat C1	T2	B2	$bla_{CTX-M-27}$	-	-	-
PV004V1F3H1E2 ★	Human H1	T2	B2	$bla_{CTX-M-27}$	-	-	-
PV004V1F3H1E3	Human H1	T2	B2	$bla_{CTX-M-27}$	-	-	-
PV004V1F4H1E1	Human H1	T2	B2	$bla_{CTX-M-27}$	-	-	-
PV004V1F3C1E1	Cat C1	T2	B2	$bla_{CTX-M-27}$	-	-	-
PV004V1F3C1E3	Cat C1	T2	B2	$bla_{CTX-M-27}$	-	-	-
PV004V1F4C1E1	Cat C1	T2	B2	$bla_{CTX-M-27}$	-	-	-
PV004V1F4C1E2	Cat C1	T2	B2	$bla_{CTX-M-27}$	-	-	-
PV004V1F4C1E3	Cat C1	T2	B2	$bla_{CTX-M-27}$	-	-	-
PV004V2F4C1E5	Cat C1	T3	B2	$bla_{CTX-M-27}$	-	-	-
PV004V2F3C1E1 ★	Cat C1	T3	B2	$bla_{CTX-M-27}$	-	-	-
PV004V0F3C1E1	Cat C1	T0	B2	$bla_{CTX-M-27}$	-	-	-
PV004V0F3C1E2 ★	Cat C1	T0	B2	$bla_{CTX-M-27}$	-	-	-
PV004V0F3C1E4	Cat C1	T0	B2	$bla_{CTX-M-27}$	-	-	-
PV004V1F3H1E1	Human H1	T2	B2	$bla_{CTX-M-27}$	-	-	-
PV004V0F3C1E3	Cat C1	T0	B2	$bla_{CTX-M-27}$	-	-	-
PV004V0F3C1E5	Cat C1	T0	B2	$bla_{CTX-M-27}$	-	-	-
PT219/3 H1F3E1	Human H1	T3	A	$bla_{CTX-M-15}$	-	-	-
PT223/0 D1F3E1	Dog 1	T0	D	$bla_{CTX-M-32}$	bla_{TEM-1}	-	-
PT223/0 D1F3E3	Dog 1	T0	D	$bla_{CTX-M-32}$	bla_{TEM-1}	-	-
PV003V1F3D1E1 ★	Dog 1	T1	B1	$bla_{CTX-M-65}$	bla_{TEM-1}	-	-
PV003V2F3D1E1 ★	Dog 1	T2	B1	$bla_{CTX-M-65}$	bla_{TEM-1}	-	-
PV003V3F3H2E2	Human H2	T3	B1	$bla_{CTX-M-65}$	bla_{TEM-1}	-	-
PV003V3F3H2E3 ★	Human H2	T3	B1	$bla_{CTX-M-65}$	bla_{TEM-1}	-	-
PV003V2F3D1E2	Dog 1	T3	B1	$bla_{CTX-M-65}$	bla_{TEM-1}	-	-
PV003V2F3D1E3	Dog 1	T2	B1	$bla_{CTX-M-65}$	bla_{TEM-1}	-	-
PV003V2F3D1E4	Dog 1	T2	B1	$bla_{CTX-M-65}$	bla_{TEM-1}	-	-
PV003V2F3D1E5	Dog 1	T2	B1	$bla_{CTX-M-65}$	bla_{TEM-1}	-	-
PV003V2F3D1E7	Dog 1	T2	B1	$bla_{CTX-M-65}$	bla_{TEM-1}	-	-
PV003V3F3D1E4	Dog 1	T3	B1	$bla_{CTX-M-65}$	bla_{TEM-1}	-	-
PV003V3F3D1E1 ★	Dog 1	T3	B1	$bla_{CTX-M-65}$	bla_{TEM-1}	-	-
PV003V2F3D1E6	Dog 1	T2	B1	$bla_{CTX-M-65}$	bla_{TEM-1}	-	-
PT214/3 D1F3E2	Dog 1	T3	B1	-	bla_{TEM-1}	-	bla_{CMY-2}
PT221/1 H2F3E2	Human H2	T1	A	$bla_{CTX-M-14}$	-	bla_{SHV-26}	-
PV001V1F3D1E1	Dog D1	T1	A	$bla_{CTX-M-14}$	bla_{TEM-1}	-	-
PV001V1F3D1E2	Dog D1	T1	A	$bla_{CTX-M-14}$	bla_{TEM-1}	-	-
PV001V1F4D1E1	Dog D1	T1	A	$bla_{CTX-M-14}$	bla_{TEM-1}	-	-
PV001V1F4D1E2	Dog D1	T1	B1	$bla_{CTX-M-14}$	bla_{TEM-1}	-	-
PT219/3 C1F3E1	Cat C1	T3	A	-	bla_{TEM-1}	bla_{SHV-12}	bla_{CMY-2}
PT224/0 D1F3E1	Dog D1	T0	B1	$bla_{CTX-M-15}$	-	-	bla_{CMY-2}
PT219/0 C1F3KE1	Cat C1	T0	A	$bla_{CTX-M-15}$	bla_{TEM-1}	-	-

Supplementary Figure S3. Dendrogram based on REP-PCR finger-printing data of 88 *Escherichia coli* carriage strains from companion animals with urinary tract infection and their cohabiting humans from Portugal. Image generated by Bionumerics (Applied Maths, Sint-Martens-Latem, Belgium) software. The first five algorithms on the strains' identification represents the household code number. Blue star represents strains selected for WGS; '-', negative for the gene.
