# Supplementary material for: Transmission dynamics of ESBL/AmpC and carbapenemase-producing Enterobacterales between companion animals and humans
Source: Front Microbiol. 2024 Sep 3;15:1432240. doi: 10.3389/fmicb.2024.1432240 (PMC11405340; doi:10.3389/fmicb.2024.1432240)
Supplement: Supplementary file 4 [file Image_4.pdf]

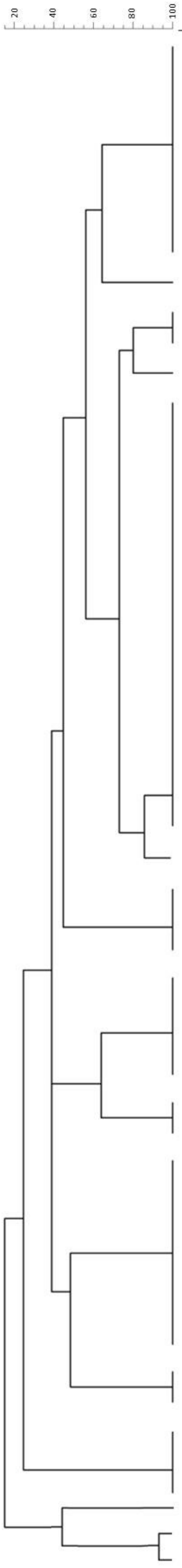

Strain code	Household member	Timepoint	Phylogroup	*bla*$_{\text{CTX-M}}$	*bla*$_{\text{TEM}}$	*bla*$_{\text{SHV}}$	*bla*$_{\text{CMY}}$	Carbapenemase
				Beta-lactamase encoding genes				
UK103/3-D1F1XE3 ★	Dog 1	T3	D	-	-	-	*bla*$_{\text{CMY-2}}$	-
UK103/2-D1F3E7.1 ★	Dog 1	T2	D	-	-	-	*bla*$_{\text{CMY-2}}$	-
UK103/3-H1F3E1 ★	Human H1	T3	D	-	-	-	*bla*$_{\text{CMY-2}}$	-
UK103/2-D1F3E8.1	Dog 1	T2	D	-	-	-	*bla*$_{\text{CMY-2}}$	-
UK103/3-D1F1XE2	Dog 1	T3	D	-	-	-	*bla*$_{\text{CMY-2}}$	-
UK103/3-D1F3E1	Dog 1	T3	D	-	-	-	*bla*$_{\text{CMY-2}}$	-
UK103/3-D1F1XE1	Dog 1	T3	D	-	-	-	*bla*$_{\text{CMY-2}}$	-
UK103/3-D1F3E2	Dog 1	T3	D	-	-	-	*bla*$_{\text{CMY-2}}$	-
UK113/1-D1F1XE2	Dog 1	T2	D	*bla*$_{\text{CTX-M-1}}$	-	*bla*$_{\text{SHV-12}}$	-	-
UK111/0-D1F3E1	Dog 1	T0	D	-	-	-	*bla*$_{\text{CMY-2}}$	-
UK111/0-D1F3E3.1	Dog 1	T0	D	-	-	-	*bla*$_{\text{CMY-2}}$	-
UK103/2-D1F3E6.1	Dog 1	T2	D	-	*bla*$_{\text{TEM-135}}$	-	*bla*$_{\text{CMY-2}}$	-
UK101/1-H1F3E9.1	Human H1	T1	D	-	-	-	*bla*$_{\text{CMY-2}}$	-
UK101/1-H1F3E11.1	Human H1	T1	D	-	-	-	*bla*$_{\text{CMY-2}}$	-
UK101/1-H1F1XE1	Human H1	T1	D	-	-	-	*bla*$_{\text{CMY-2}}$	-
UK101/1-H1F3E1	Human H1	T1	D	-	-	-	*bla*$_{\text{CMY-2}}$	-
UK101/1-H1F3E3	Human H1	T1	D	-	-	-	*bla*$_{\text{CMY-2}}$	-
UK101/1-H1F3E4	Human H1	T1	D	-	-	-	*bla*$_{\text{CMY-2}}$	-
UK101/1-H1F3E5 ★	Human H1	T1	D	-	-	-	*bla*$_{\text{CMY-2}}$	-
UK101/1-H1F3E6	Human H1	T1	D	-	-	-	*bla*$_{\text{CMY-2}}$	-
UK101/2-D1F1XE2	Dog D1	T2	D	-	-	-	*bla*$_{\text{CMY-2}}$	-
UK101/1-H1F3E3	Human H1	T1	D	-	-	-	*bla*$_{\text{CMY-2}}$	-
UK101/2-D1F1XE1	Dog D1	T2	D	-	-	-	*bla*$_{\text{CMY-2}}$	-
UK101/2-D1F1XE3 ★	Dog D1	T2	D	-	-	-	*bla*$_{\text{CMY-2}}$	-
UK101/2-D1F1XE4	Dog D1	T2	D	-	-	-	*bla*$_{\text{CMY-2}}$	-
UK101/1-H1F3E2	Human H1	T1	D	-	-	-	*bla*$_{\text{CMY-2}}$	-
UK101/1-H1F3E7	Human H1	T1	D	-	-	-	*bla*$_{\text{CMY-2}}$	-
UK103/3-H1F3E2	Human H1	T3	D	-	-	-	*bla*$_{\text{CMY-2}}$	-
UK105/0-D1F3E8.1	Dog D1	T1	D	-	-	-	*bla*$_{\text{CMY-2}}$	-
UK105/0-D1F3E6.1	Dog D1	T1	D	-	-	-	*bla*$_{\text{CMY-2}}$	-
UK105/0-D1F3E7.1	Dog D1	T1	D	-	-	-	*bla*$_{\text{CMY-2}}$	-
UK108/1-D1N3E1	Dog D1	T2	D	-	-	-	*bla*$_{\text{CMY-2}}$	-
UK108/1-D1F3E1	Dog D1	T2	D	-	-	-	*bla*$_{\text{CMY-2}}$	-
UK108/0-D1F1XE2	Dog D1	T0	D	-	-	-	*bla*$_{\text{CMY-2}}$	-
UK108/1-D1F4E3	Dog D1	T2	D	-	-	-	*bla*$_{\text{CMY-2}}$	-
UK108/1-D1F3E2	Dog D1	T2	D	-	-	-	*bla*$_{\text{CMY-2}}$	-
UK111/1-D1F4E1W ★	Dog D1	T2	A	*bla*$_{\text{CTX-M-15}}$	-	-	*bla*$_{\text{CMY-2}}$	*bla*$_{\text{NDM-5}}$
UK111/1-D1F1XE2	Dog D1	T2	A	*bla*$_{\text{CTX-M-15}}$	-	-	*bla*$_{\text{CMY-2}}$	*bla*$_{\text{NDM-5}}$
UK111/0-D1F3UE1 ★	Dog D1	T0	D	*bla*$_{\text{CTX-M-15}}$	-	-	-	*bla*$_{\text{NDM-5}}$
UK111/1-D1F1TE3 ★	Dog D1	T2	D	*bla*$_{\text{CTX-M-15}}$	-	-	-	*bla*$_{\text{NDM-5}}$
UK111/1-D1F4E2	Dog D1	T2	D	*bla*$_{\text{CTX-M-15}}$	-	-	-	*bla*$_{\text{NDM-5}}$
UK111/1-D1F3E4W	Dog D1	T2	D	*bla*$_{\text{CTX-M-15}}$	-	-	-	*bla*$_{\text{NDM-5}}$
UK111/1-D1F3E1W	Dog D1	T2	D	*bla*$_{\text{CTX-M-15}}$	-	-	-	*bla*$_{\text{NDM-5}}$
UK111/1-D1F1CE4	Dog D1	T2	D	*bla*$_{\text{CTX-M-15}}$	-	-	-	*bla*$_{\text{NDM-5}}$
UK111/1-D1F1CE2	Dog D1	T2	D	*bla*$_{\text{CTX-M-15}}$	-	-	-	*bla*$_{\text{NDM-5}}$
UK111/1-D1N3E1 ★	Dog D1	T2	D	*bla*$_{\text{CTX-M-15}}$	-	-	-	*bla*$_{\text{NDM-5}}$
UK113/0-D1F3E2W	Dog D1	T0	B1	-	-	*bla*$_{\text{SHV-12}}$	-	-
UK113/0-D1F3E4W	Dog D1	T0	B1	-	-	*bla*$_{\text{SHV-12}}$	-	-
UK107/0-D1F3KE4	Dog D1	T0	A	-	*bla*$_{\text{TEM-135}}$	-	*bla*$_{\text{CMY-2}}$	-
UK107/0-D1F3KE5	Dog D1	T0	A	-	*bla*$_{\text{TEM-135}}$	-	*bla*$_{\text{CMY-2}}$	-
UK107/0-D1F3KE6	Dog D1	T0	A	-	*bla*$_{\text{TEM-135}}$	-	*bla*$_{\text{CMY-2}}$	-
UK108/0-D1F3E1	Dog D1	T0	B2	-	-	-	*bla*$_{\text{CMY-2}}$	-
UK201/1-D1F1XE1	Dog D1	T2	D	*bla*$_{\text{CTX-M-15}}$	-	-	*bla*$_{\text{CMY-2}}$	-
UK201/2-D1F3E1	Dog D1	T3	A	*bla*$_{\text{CTX-M-1}}$	*bla*$_{\text{TEM-1}}$	-	-	-

Supplementary Figure S4. Dendrogram based on REP-PCR finger-printing data of 54 *Escherichia coli* carriage strains from companion animals with skin and soft tissue or urinary tract infections and their cohabiting humans from the United Kingdom. Image generated by Bionumerics (Applied Maths, Sint-Martens-Latem, Belgium) software. The first five algorithms on the strains' identification represents the household code number. Blue star represents strains selected for WGS; orange stars identified NDM-5 producing strains from household PT111 previously described by our group (Menezes, S.-M. Frosini, *et al.*, 2023; underline strains code refers to nasal swab isolates, otherwise are faecal isolates; '-', negative for the gene.
